# Machine learning-based prediction of short- and long-term mortality for shared decision-making in older hip fracture patients: the Dutch Hip Fracture Audit algorithms in 74,396 cases

**DOI:** 10.2340/17453674.2025.44248

**Published:** 2025-07-07

**Authors:** Hidde DIJKSTRA, Cathleen S PARSONS, Hanne-Eva VAN BREMEN, Hanna C WILLEMS, Anne A H DE HOND, Barbara C VAN MUNSTER, Job N DOORNBERG, Jacobien H F OOSTERHOFF

**Affiliations:** 1 Department of Orthopaedic Surgery, University Medical Centre Groningen, University of Groningen; 2 University Center for Geriatric Medicine, University of Groningen, University Medical Center Groningen, Groningen; 3 Department of Engineering Systems & Services, Faculty of Technology Policy and Management, Delft University of Technology, Delft; 4 Amsterdam Bone Center, Movement Sciences Amsterdam, Amsterdam; 5 Dutch Institute for Clinical Auditing, Leiden; 6 Amsterdam University Medical Centers, location Academic Medical Center, Internal Medicine and Geriatrics, University of Amsterdam, Amsterdam; 7 Amsterdam Public Health Research Institute, Amsterdam;; 8 Julius Centre for Health Sciences and Primary Care, University Medical Center, Utrecht; the Netherlands

## Abstract

**Background and purpose:**

Treatment-related shared decision-making (SDM) in older adults with hip fractures is complex due to the need to balance patient-specific factors such as life goals, frailty, and surgical risks. It includes considerations such as prognosis and decisions concerning whether to operate or not on frail, life-limited patients. We aimed to develop machine learning (ML)-driven prediction models for short- and long-term mortality in a large cohort of patients with hip fractures.

**Methods:**

In this national registry-based retrospective cohort study, patients aged ≥ 70 years registered in the nationwide Dutch Hip Fracture Audit from 2018–2023 were included. Predictive variables were selected based on the literature and/or clinical relevance. 6 ML algorithms, including logistic regression, were trained with internal cross-validation and evaluated on discrimination (c-statistic), sensitivity, specificity, calibration, and interpretability.

**Results:**

74,396 patients (median age 84, IQR 78–89; 68% female) were analyzed. Most patients lived at home (69%) and high malnutrition risk was seen in 10%. 18% had dementia. Mortality rates were 9.1% (30-day), 15% (90-day), and 26% (1-year). Logistic regression performed comparably to other algorithms, but was chosen as the preferred algorithm due to its superior interpretability (c-statistic: 30-day 0.82, 90-day 0.81, 1-year 0.80).

**Conclusion:**

We developed and validated ML algorithms, including logistic regression, for mortality prediction in older hip fracture patients with adequate performance. This information may inform SDM.

Frail older patients with hip fractures often do not live independently and face high morbidity and mortality rates [1]. Hip fractures also significantly impact health-related quality of life and further reduce life expectancy, presenting a substantial personal and public health concern [2]. Surgical treatment, such as fracture fixation or arthroplasty, has traditionally been the preferred treatment. In general hip fracture populations aged 60 years and older, 1-year mortality rates of up to 20% have been observed [3]. Recent studies suggest that nonoperative, palliative treatment may be appropriate for carefully selected frail patients with limited life expectancies, focusing on care goals, pain management, and comfort [4-6]. Although still highly debated, these findings highlight the importance of considering both operative and nonoperative management in the frailest patients with hip fractures [4,5].

The optimal treatment for hip fracture patients depends on fracture configuration and overall health status, as well as on the patient’s and caregiver’s priorities in decision-making, including life and treatment goals [1,7]. Shared decision-making (SDM), weighing pros and cons of each treatment involving patients, families, and healthcare professionals, is crucial. Machine learning (ML) models offer individualized mortality predictions, often important to patients and linked to other aspects such as discharge destination, aiding SDM and reducing decision bias [8]. Moreover, predicting mortality may help identify patients with limit life expectancies, guiding which patient may benefit from arthroplasty srugery (hemi- or total hip arthroplasty), internal fixation (e.g. a sliding hip screw or cancellous screws) or nonoperative management. While many ML models exist, they often rely on variables that are not widely available, hindering validation or their potential use in different settings such as in nursing homes. Moreover, the added value of ML for mortality prediction has been explored in only a few countries, with the Dutch population not yet studied. Using data from the Dutch Hip Fracture Audit (DHFA), we aimed to develop ML-driven models for short-term (30- and 90-day) and longer-term (1-year) mortality prediction in Dutch hip fracture patients.

## Methods

### Study design and participants

Data for this retrospective cohort study was obtained from the DHFA, a nationwide registry for hip fracture patients in the Netherlands [9]. More than 95% of all Dutch hospitals are included in this registry [10]. Registered hip fracture patients treated in these 66 hospitals between January 1, 2018 and December 31, 2023 and aged 70 years or older were included. The registry includes patients undergoing operative and nonoperative treatment and excludes patients with periprosthetic or pathologic fractures. Data from hospitals is submitted via a secure survey system, either completed directly by clinicians or through batch data processing. These submissions undergo validation processes, ensuring data completeness and accuracy. Previous verifications of this hip fracture registry have consistently demonstrated high data completeness > 95% and quality [10,11]. Mortality data was sourced from the Dutch Vektis Data Institute, which aggregates data from health insurance reimbursements. A trusted party combined the DHFA and Vektis data using social security numbers, resulting in an anonymized dataset provided to the researchers. As a result, patients with missing data on their social security number were excluded. Data was preprocessed based on previous studies using the same dataset [1,10].

### Primary outcomes

The primary outcomes of this study were 30-day, 90-day, and 1-year mortality, based on the day of hospital admittance serving as starting point for this data.

### Explanation of candidate input variables

First, based on variable availability in the registry (those with less than 30% missing), we selected 11 potential predictors. We excluded fracture type based on not being available for patients treated exclusively in nursing homes, as radiographs for fracture type determination are not typically performed in nursing homes, limiting the model’s applicability in this setting. Second, we selected 8 potential predictors based on the literature: (i) age (continuous); (ii) sex (binary); (iii) American Society of Anesthesiologists (ASA) Classification (I–V); (iv) pre-fracture living status (living in nursing homes or not); (v) pre-fracture functional status (mobile without mobility aid, using 1 aid, using 2 aids/frame, mobile inside but not outside, no functional mobility); (vi) dementia (binary); (vii) risk of malnutrition (no risk, slight/medium risk, and high risk); (viii) fracture side (binary) [12-15]. We chose to include 2 additional variables based on clinical relevance: (i) daily living dependency following the Katz Index of Independence in Activities of Daily Living (KATZ-ADL) (independent [score 0], dependent [score 1–3], and more dependent [score 4–6]); and (ii) osteoporosis (binary). Simultaneously, all variables were assessed on clinical relevance by our research team, which includes all relevant medical disciplines. Finally, we examined inter-variable relationships through a heat map (using Pearson correlation coefficients) and chi-square tests for all variables, and we included the abovementioned 10 predictors.

Presence of dementia and osteoporosis were confirmed if diagnosed by a general practitioner or documented in the treating hospital’s records. Dementia was included as predictor as it has been associated with malnutrition, functional outcomes, and rehabilitation success [16]. The pre-fracture daily living dependency was calculated using the KATZ-ADL scale, which has been previously validated for the Dutch language [17]. The risk of malnutrition was categorized as high, medium, or no risk according to the Short Nutritional Assessment Questionnaire (SNAQ) or the Malnutrition Universal Screening Tool (MUST) [18,19]. The pre-fracture mobility was determined using the fracture mobility score, which has previously been validated in patients with hip fractures [20]. All predictors were obtained from the DHFA registry.

### Missing data, model development, hyperparameter tuning, and performance assessment

Handling missing data, model development, hyperparameter tuning, and explanation and model performance assessment are described in detail in Supplementary Tables I–IV. The primary performance metric of interest was discrimination (c-statistic), which represents the area under the receiver operating curve (ROC). This value ranges from 0.50 (random discrimination) to 1.0 (perfect discrimination), thus indicating how well the prediction model distinguishes between patients who experience the outcome (e.g., mortality) and those who do not [21]. Other performance metrics of particular interest were calibration and sensitivity. Calibration compares predicted probabilities (x-axis) with observed probabilities (y-axis) using calibration curves characterized by the calibration slope and intercept. A slope of 1 and intercept of 0 indicates perfect calibration [22]. Sensitivity is the proportion of correctly classified positive observations out of all actual positives, with 100% sensitivity indicating that all positive observations were correctly identified. When selecting the superior performing algorithm for a specific outcome (e.g., 30-day mortality), the combination of performance metrics and model interpretability were taken into account.

### Statistics

Data pre-processing and analysis was performed using Python version 3.11.4 (https://www.python.org/downloads/release/python-3114/). Logistic regression was included as a simpler supervised ML algorithm for comparison with more complex algorithms: Extreme Gradient Boosting (XGBoost), Random Forest, Linear Support vector machine, Elastic net Penalized logistic regression (more advanced, regularized version of logistic regression), K-Nearest Neighbors, and logistic regression. A short description of each algorithm is provided in Supplementary Table II. The following packages were used: pandas (version 1.5.3; https://pandas.pydata.org/pandas-docs/version/1.5.3/), matplotlib (version 3.7.1; https://matplotlib.org/3.7.1/), seaborn (version 0.12.2; https://seaborn.pydata.org/archive/0.12/index.html), numpy (version 1.24.3; https://pypi.org/project/numpy/), scikit-learn (version 1.4.2; https://pypi.org/project/scikit-learn/1.4.2/), and xgboost (version 2.0.3; https://pypi.org/project/xgboost/). Categorical variables were described as absolute numbers with frequencies, and continuous variables as medians with interquartile ranges (IQR). The model performance metrics were calculated with 95% confidence intervals (CI).

### Data safety and guideline adherence

Our Machine Learning consortium [8,23] adhered to the “Policy on Use and Sharing of Data Collected by World Health Organization (WHO) in Member States Outside the Context of Public Health Emergencies” of the WHO for safe multicenter data exchange and analysis [24].

This study was conducted in accordance with the Transparent Reporting of Multivariable Prediction Models for Individual Prognosis or Diagnosis Guideline (TRIPOD+AI Statement), the Prediction model Risk of Bias Assessment Tool (PROBAST+AI), and the JMIR Guidelines for Developing and Reporting Machine Learning Predictive Models in Biomedical Research [25-27].

### Ethics, registration, funding, use of AI, and disclosures

The data was anonymized. Data was retrieved from Dutch hospitals in the DHFA, after which a trusted third party pseudonymized the data. The scientific committee of the DHFA approved this research using DHFA data, and data was not deemed subject to the Medical Research Involving Human Subjects Act in compliance with Dutch regulations. The Dutch law allows the use of electronic health records for research purposes under certain conditions. According to this legislation, neither obtaining informed consent from patients nor approval by a medical ethics committee is obligatory for this type of observational studies containing no directly identifiable data. This work was supported by the Anna Foundation | NOREF. One of the authors (HD) certifies that he received an amount less than US$10,000 from this Foundation. Generative AI tools were used for editing the manuscript. Each author certifies that he or she has no commercial associations (e.g., consultancies, stock ownership, equity interest, patent/licensing arrangements, etc.) that might pose a conflict of interest in relation to the submitted article. Complete disclosure of interest forms according to ICMJE are available on the article page, doi: 10.2340/17453674.2025.44248

## Results

### Study population

This study included 74,396 patients. The median age was 84 years (IQR 78–89), and most patients were female (68%). The 30-day mortality rate was 9.1% (n = 6,746), the 90-day mortality rate was 15% (n = 10,732), and the 1-year mortality rate was 26% (n = 16,202), calculated from the outcome-specific datasets (Table 1).

**Table 1:** Patient, fracture characteristics and mortality rates (N = 74,396 ^a^). Values are count (%) unless otherwise specified

Age, median (IQR)	84 (78–89)
Age	
70–79	23,451 (32)
80–89	35,323 (47)
≥ 90	15,622 (21)
Sex	
Female	50,794 (68)
Male	23,498 (32)
ASA classification	
I	1,873 (2.5)
II	21,650 (29)
III	40,054 (54)
IV	5,204 (7.0)
V	39 (<0.1)
Pre-fracture living situation	
Not institutionalized	51,324 (69)
Institutionalized	13,181 (18)
Pre-fracture functional status	
Mobile without mobility aid	30,189 (41)
Mobile using 1 mobility aid	5,794 (7.8)
Mobile using 2 mobility aids or frame	25,555 (34)
Not mobile outside without help	6,788 (9.1)
No functional use of lower extremities	1,256 (1.7)
Dementia	
Yes	13,601 (18)
No	50,615 (68)
Daily living dependency	
Independent (KATZ6-ADL 0)	36,543 (49)
Dependent (KATZ6-ADL 1–3)	19,525 (26)
More dependent (KATZ6-ADL 4–6)	14,987 (20)
Risk of malnutrition	
No risk (SNAQ 0 or MUST 0)	53,209 (72)
Slight/medium risk (SNAQ 1–2 or MUST 1)	8,295 (11)
High risk (SNAQ ≥ 3, MUST ≥ 2)	7,655 (10)
Osteoporosis	
Yes	8,060 (11)
No	55,234 (74)
Fracture side	
Left	38,460 (52)
Right	35,779 (48)
Fracture type	
Femoral neck fracture, nondisplaced	11,762 (16)
Femoral neck fracture, displaced	28,266 (38)
Trochanteric fracture, AO–A1	9,656 (13)
Trochanteric fracture, AO–A2	14,425 (19)
Trochanteric fracture, AO–A3	4,296 (5.8)
Subtrochanteric fracture	2,820 (3.8)
Treatment strategy	
Treated surgically	71,306 (96)
Treated conservatively	3,090 (4.2)
Mortality rates (after hospital admission)**^b^**	
30-day	6,746 (9.1)
90-day	10,732 (15)
1-year	16,202 (26)

IQR = interquartile range. ASA = American Society of Anesthesiolo-gists classification. KATZ6-ADL = Katz Index of Independence inActivities of Daily Living. SNAQ = Short Nutritional AssessmentQuestionnaire. MUST = Malnutrition Universal Screening Tool. AO =Arbeitsgemeinschaft für Osteosynthesefragen Classification systemfor proximal femur fractures.

a Missing values: sex (n = 104, 0.1%), ASA class (n = 5,576, 7.5%),pre-fracture living situation (n = 9,891, 13%), pre-fracture functionalstatus (n = 4,814, 6.5%), dementia (10,180, 14%), daily livingdependency (n = 3,341, 4.5%), risk of malnutrition (n = 5,237, 7.0%), osteoporosis (n = 11,102, 15%), fracture side (n = 146, 0.2%), fracture type (n = 3,171, 4.3%).

b Percentages are based on the incidence calculated from eachoutcome-specific dataset.

### Performance of ML algorithms predicting 30-day mortality

In the training set (n = 59,561), the 6 algorithms showed c-statistics ranging from 0.81–0.84, calibration slopes ranging from 0.95–1.24 and intercepts ranging from –0.02 to 0.00 (Supplementary Table V). Sensitivity ranged from 0.73–0.87, specificity ranged from 0.56–0.74, and the PR-AUC from 0.30–0.41. Precision (PPV), F1 scores, and Brier scores for these analyses and the analyses below can be found in Supplementary Tables VI, IX, XII, XV, XVIII, and XXI.

**Figure 1:**
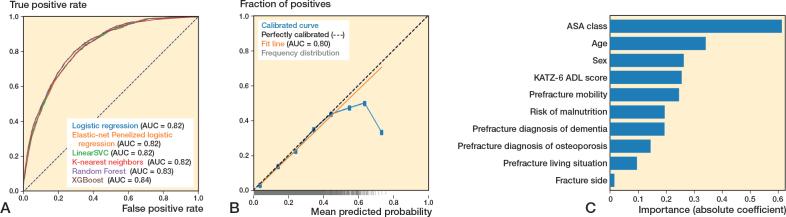
(A) Receiver operating characteristic curves per algorithm showing the area under the curve (AUC) for each algorithm, for the outcome 30-day mortality. (B) Calibration curve for the logistic regression algorithm for the outcome 30-day mortality. (C) Variable importance for 30-day mortality using the preferred algorithm (preferred based on transparency), logistic regression. Absolute coefficient = the absolute values of the standardized regression coefficients (|β|) from a logistic regression model, making the importances comparable across the variables.

In the test set (n = 14,480), the 6 algorithms showed c-statistics ranging from 0.81–0.83 (Figure 1A, Table 2). The calibration slopes ranged from 0.98–1.12 and calibration intercepts ranged from –0.02 to 0.00 (Figure 1B; logistic regression). Sensitivity ranged from 0.73–0.89, specificity ranged from 0.55–0.74, and the PR-AUC ranged from 0.30–0.44 (used threshold 0.10) (Supplementary Tables VI, IX, XII, XV, XVIII, and XXI).

**Table 2:** Performance of machine learning algorithms in predicting 30-day, 90-day, and 1-year mortality in the test set after 1,000 bootstrapping iterations using different thresholds for each outcome: 0.10 (30-day mortality), 0.15 (90-day mortality), and 0.25 (1-year mortality)

Algorithm	Extreme	Random	Linear Support	Elastic-Net Penalized	K-Nearest-	Logistic
	Gradient Boosting	Forest	Vector Machine	Logistic Regression	Neighbour	regression^a^
Metric (30-day mortality)						
c-statistic	0.81 (0.81 to 0.82)	0.83 (0.82 to 0.84)	0.82 (0.81 to 0.83)	0.81 (0.80 to 0.82)	0.82 (0.81 to 0.83)	0.82 (0.81 to 0.83)
Calibration slope	0.98 (0.94 to 1.03)	1.12 (1.06 to 1.19)	0.93 (0.87 to 0.99)	1.06 (1.02 to 1.11)	1.00 (0.94 to 1.06)	0.97 (0.90 to 1.03)
Calibration intercept	0.00 (0.00 to 0.01)	–0.02 (–0.02 to –0.01)	0.00 (0.00 to 0.01)	–0.01 (–0.02 to 0.00)	0.00 (0.00 to 0.01)	–0.01 (–0.01 to 0.00)
Sensitivity	0.78 (0.76 to 0.79)	0.78 (0.76 to 0.81)	0.73 (0.71 to 0.76)	0.89 (0.87 to 0.90)	0.76 (0.73 to 0.78)	0.77 (0.74 to 0.79)
Specificity	0.69 (0.69 to 0.70)	0.72 (0.71 to 0.72)	0.74 (0.74 to 0.75)	0.55 (0.55 to 0.56)	0.73 (0.72 to 0.73)	0.72 (0.71 to 0.72)
PR-AUC	0.44 (0.42 to 0.46)	0.33 (0.31 to 0.36)	0.30 (0.28 to 0.33)	0.42 (0.39 to 0.44)	0.30 (0.28 to 0.33)	0.30 (0.28 to 0.33)
Metric (90-day mortality)						
c-statistic	0.81 (0.81 to 0.82)	0.81 (0.81 to 0.82)	0.81 (0.80 to 0.82)	0.81 (0.80 to 0.82)	0.80 (0.79 to 0.81)	0.81 (0.80 to 0.82)
Calibration slope	0.98 (0.94 to 1.03)	1.14 (1.09 to 1.18)	0.96 (0.92 to 1.01)	1.06 (1.02 to 1.11)	1.04 (0.99 to 1.09)	0.98 (0.94 to 1.03)
Calibration intercept	0.00 (0.00 to 0.01)	–0.02 (–0.03 to –0.02)	0.00 (0.00 to 0.01)	–0.01 (–0.02 to 0.00)	0.00 (0.00 to 0.01)	0.00 (–0.01 to 0.00)
Sensitivity	0.78 (0.76 to 0.79)	0.80 (0.79 to 0.82)	0.76 (0.74 to 0.77)	0.77 (0.75 to 0.78)	0.78 (0.76 to 0.80)	0.88 (0.87 to 0.90)
Specificity	0.69 (0.69 to 0.70)	0.67 (0.66 to 0.68)	0.71 (0.70 to 0.72)	0.70 (0.69 to 0.71)	0.69 (0.68 to 0.70)	0.55 (0.55 to 0.56)
PR-AUC	0.44 (0.42 to 0.46)	0.44 (0.42 to 0.46)	0.41 (0.39 to 0.44)	0.42 (0.39 to 0.44)	0.40 (0.38 to 0.42)	0.42 (0.39 to 0.44)
Metric (1-year mortality)						
c-statistic	0.81 (0.80 to 0.81)	0.80 (0.80 to 0.81)	0.80 (0.79 to 0.81)	0.81 (0.80 to 0.82)	0.80 (0.79 to 0.81)	0.80 (0.79 to 0.81)
Calibration slope	0.99 (0.96 to 1.02)	1.10 (1.07 to 1.14)	0.98 (0.94 to 1.01)	1.06 (1.02 to 1.11)	1.02 (0.99 to 1.06)	0.99 (0.95 to 1.02)
Calibration intercept	0.00 (–0.01 to 0.01)	–0.03 (–0.04 to –0.02)	0.01 (0.00 to 0.01)	–0.01 (–0.02 to 0.00)	0.00 (0.00 to 0.01)	0.00 (–0.01 to 0.01)
Sensitivity	0.77 (0.75 to 0.78)	0.78 (0.77 to 0.79)	0.74 (0.73 to 0.76)	0.53 (0.51 to 0.55)	0.77 (0.75 to 0.78)	0.75 (0.74 to 0.77)
Specificity	0.68 (0.68 to 0.69)	0.67 (0.66 to 0.68)	0.71 (0.70 to 0.72)	0.86 (0.85 to 0.87)	0.69 (0.68 to 0.70)	0.70 (0.69 to 0.71)
PR-AUC	0.58 (0.57 to 0.60)	0.58 (0.56 to 0.60)	0.56 (0.54 to 0.58)	0.42 (0.39 to 0.44)	0.56 (0.54 to 0.58)	0.56 (0.55 to 0.58)

PR-AUC = Area Under the Curve of the Precision Recall Curve.

a Logistic regression algorithm was the preferred predictive algorithm for 30-day, 90-day, and 1-year mortality prediction in the test set.

### Performance of ML algorithms predicting 90-day mortality

For 90-day mortality, in the training set (n = 57,920), the 6 algorithms showed c-statistics ranging from 0.81–0.83, calibration slopes ranging from 0.96–1.20, and intercepts ranging from –0.03 to 0.01 (Supplementary Table V). Sensitivity ranged from 0.74–0.87, specificity ranged from 0.56–0.72, and the PR-AUC ranged from 0.10–0.48 (Supplementary Tables VII, X, XIII, XVI, XIX, and XXII).

**Figure 2:**
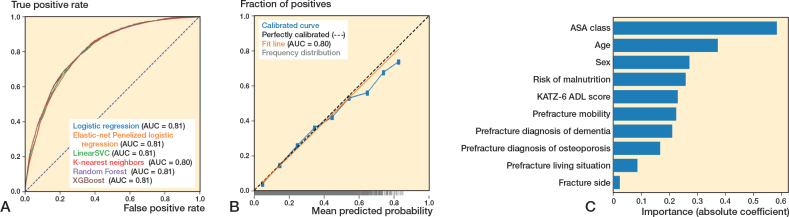
(A) Receiver operating characteristic curves per algorithm showing the area under the curve (AUC) for each algorithm, for the outcome 90-day mortality. (B) Calibration curve for the logistic regression algorithm for the outcome 90-day mortality. (C) Variable importance for 90-day mortality using the preferred algorithm (preferred based on transparency), logistic regression. Absolute coefficient = the absolute values of the standardized regression coefficients (|β|) from a logistic regression model, making the importances comparable across the variables.

In the test set (n = 14,489), the 6 algorithms showed c-statistics ranging from 0.80–0.81 (Figure 2A, Table 2). The calibration slopes ranged from 0.96–1.14 and calibration intercepts ranged from –0.02 to 0.00 (Figure 2B; logistic regression). Sensitivity ranged from 0.76–0.88 and specificity ranged from 0.55–0.71, and the PR-AUC ranged from 0.40–0.44 (used threshold 0.15) (Supplementary Tables VII, X, XIII, XVI, XIX, and XXII).

### Performance of ML algorithms predicting 1-year mortality

For 1-year mortality, in the training set (n = 50,241), the 6 algorithms showed c-statistics ranging from 0.80–0.82, calibration slopes ranging from 0.98–1.16, and intercepts ranging from –0.04 to 0.00 (Supplementary Table V). Sensitivity ranged from 0.74–0.87 and specificity ranged from 0.40–0.70, and the PR-AUC ranged from 0.41–0.61 (Supplementary Tables VIII, XI, XIV, XVII, XX, and XXIII).

**Figure 3:**
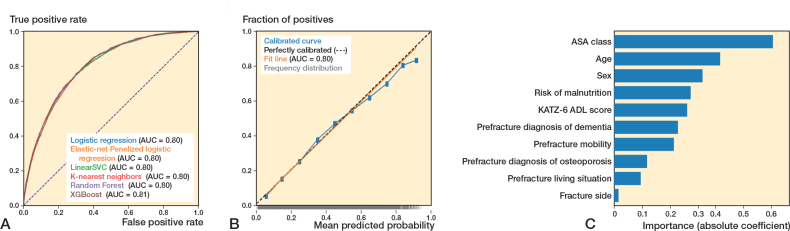
(A) Receiver operating characteristic curves per algorithm showing the area under the curve (AUC) for each algorithm, for the outcome 90-day mortality. (B) Calibration curve for the logistic regression algorithm for the outcome 90-day mortality. (C) Variable importance for 1-year mortality using the preferred algorithm (preferred based on transparency), logistic regression. Absolute coefficient = the absolute values of the standardized regression coefficients (|β|) from a logistic regression model, making the importances comparable across the variables.

In the test set (n = 12,561), the 6 algorithms showed c-statistics ranging from 0.80–0.81 (Figure 3A, Table 2). The calibration slopes ranged from 0.99–1.10 and calibration intercepts ranged from –0.03 to 0.00 (Figure 3B; logistic regression), sensitivity ranged from 0.53–0.78, specificity ranged from 0.67–0.86, and the PR-AUC ranged from 0.42–0.58 (used threshold 0.25) (Supplementary Tables VIII, XI, XIV, XVII, XX, and XXIII).

### Preferred algorithms across all outcomes: Dutch Hip Fracture Audit algorithms

All algorithms showed similar performance across outcomes, with XGBoost and logistic regression performing comparably in terms of c-statistic, calibration, and sensitivity, the main metrics of interest. The logistic regression algorithm was chosen as the preferred algorithm, because of its interpretability (i.e., transparency). ASA class, age, and sex were the strongest predictors in the models (Figures 1C–3C). The confusion matrix showing the number of correct and incorrect predictions for these models are shown in Supplementary Figures I–III, from which the positive predictive value (PPV) and negative predictive value (NPV) can also be derived. In Supplementary Table IV an example prediction for 30-day mortality using the preferred algorithm is included, to provide probabilistic rather than binary risk scores.

## Discussion

Our study is the first to develop and evaluate short- and long-term mortality probability estimations exploring ML including logistic regression, specific to the Dutch population, using a comprehensive national registry-based dataset. We used a large comprehensive national registry database, and the prediction models showed adequate performance, demonstrated by good discrimination, calibration, and sensitivity, the measures of particular interest. Moreover, this study prioritized the explainability of the algorithms, which is crucial for clinical understanding and implementation.

Recent research has increasingly focused on predicting mortality in older adults with hip fractures. A systematic review identified 27 studies employing ML for patients with hip fractures to predict several outcomes, being most often mortality [23]. Liu et al. reported in their review a pooled c-statistic of 0.76 (training sets) and 0.84 (test sets) across 8 studies for mortality prediction [14]. Our findings yielded comparable c-statistics for 30-day (0.82), 90-day (0.81), and 1-year (0.80) mortality predictions using logistic regression in test sets. Moreover, Liu et al. demonstrated that ML models outperform traditional clinical scoring systems, such as the Nottingham Hip Fracture Score (NHFS), which is commonly validated in the Dutch population [14]. The Almelo Hip Fracture Score (AHFS), a Dutch variant of the NHFS, initially showed a c-statistic of 0.82 but dropped to 0.70 when externally validated [13,28]. We investigated ML algorithms using a national cohort including over 95% of Dutch hospitals. Logistic regression was chosen as the preferred model for its interpretability, despite comparable performance to more complex algorithms. Overall, our findings indicate that both complex ML algorithms and simpler ML techniques like logistic regression demonstrate adequate but comparable predictive performance in Dutch patients. Moreover, by using variables that are readily available, the preferred model may be easily validated and usable in different settings and countries in the future.

A recent systematic review identified high-quality predictors for 30-day mortality: age, ASA score > 3, being male, institutional residence, and metastatic cancer [12]. Our study included the same predictors, with the exception of metastatic cancer. Additionally, we incorporated other predictors: dementia, malnutrition, fracture side, and pre-fracture mobility, which are linked to increased risk of dying within 1 year [12].

### Strengths

First, this was the first study to apply ML for postoperative mortality prediction in Dutch older adults with hip fractures, using a large national cohort representing over 95% of Dutch hospitals, enhancing generalizability for Dutch patients [10]. Second, the study emphasized explainable artificial intelligence, making the model more understandable for clinicians by providing insights into underlying theory of the algorithms, algorithm training and internal validation, and information on variable importance. Third, this study used meaningful predictive variables, such as ADL dependency, malnutrition risk, and pre-fracture functional status, which are important for geriatric rehabilitation and health-related quality of life [2].

### Limitations

First, this study may be subject to registration bias, with up to 15% missing data depending on the variable, though registry quality has improved in recent years [10]. Second, including additional variables, such as psychosocial factors (e.g., loneliness) [13], could have improved the model. Adding such variables aligns with the warranted holistic approach for the patient population. Third, the prevalence of osteoporosis and dementia in this study may be underestimated [29]. Nonetheless, we anticipated that the degree of underestimation is limited and unlikely to have introduced substantial noise into the analysis. Future validation studies should account for this by evaluating the incidence and the predictive importance of these variables. Fourth, although the models performed adequately (and slightly better than existing risk scores), calibration, sensitivity, and specificity were not perfect, which should be considered for implementation and validation. Additionally, the model should be validated in a controlled or pilot setting before full clinical implementation to ensure its performance meets clinical expectations. Moreover, this study encountered class imbalance, which was adequately addressed by optimizing thresholds and using metrics sensitive to imbalance, such as c-statistic and PR-AUC. Lastly, the predictive performance can differ among populations, even in the same country. The generalizability to other countries may be limited. Therefore, prospective, local validation studies (e.g., regional, hospital specific) are needed.

### Clinical implications

By using predictive variables also available in nursing homes, we consider the DHFA algorithms usable outside the hospital. The preferred algorithm offers prognostic information for health professionals to integrate with the patient’s specific situation and preferences. Whether to share these probabilities with the patient should depend on individual factors, such as health literacy. A high 30-day mortality estimate (e.g., ~90%) may indicate lower life expectancy, suggesting that nonoperative, palliative management might be more appropriate. However, the decision should not be based solely on this prediction. It should be made in conjunction with the patient’s individual life and treatment goals, alongside other considerations. Conversely, a low estimate may support the decision for surgery, still depending on individual circumstances [4]. The clinical value of middle-range probabilities (e.g., 50–60%) may be unclear and should be assessed within the SDM context before implementation. Further, clinical acceptance and usability by health professionals and patients should also be evaluated before implementation. Second, although the DHFA algorithms are broadly applicable to the Dutch population, prospective evaluation is needed due to inter-hospital differences in care quality impacting mortality outcomes; the ongoing data collection by DICA-DHFA enables future validation. Third, the DHFA algorithm providing probabilities for 1-year mortality may identify patients who would benefit from osteoporosis assessment and medication. When a high 1-year mortality is predicted, the osteoporosis medication might be discontinued. This aligns with Dutch guidelines recommending consideration of osteoporosis treatment for patients with a life expectancy of over 1 year [30]. Lastly, it is important to note that this study included frail patients treated nonoperatively, who have lower life expectancies compared with those treated surgically [7]. Further, the aim of this study was to develop a prediction model to support SDM prior to the selection of a treatment approach. Therefore, excluding patients treated conservatively would not align with this objective, as treatment strategy information is not available at this stage.

### Conclusion

We developed and validated ML algorithms, including logistic regression, for mortality prediction in older hip fracture patients with adequate performance.

*In perspective,* future research should prioritize validating and prospectively evaluating DHFA algorithms in international and local populations. Ultimately, this information, alongside other important considerations, will support the SDM process, leading to a more personalized, data-driven care strategy for this frail patient population.

### Supplementary data

Tables I–XXII and Figure I–III are available as supplementary data on the article page, doi: 10.2340/17453674.2025.44248

## Supplementary Material



## Notes

HD: conceptualization, methodology, data curation, writing—original draft, writing—review and editing. CP: methodology, formal analysis, data curation, visualization, writing—review and editing. HvB: supporting data curation, writing—review and editing. HW: writing—review and editing. AH: supporting methodology and formal analysis, writing—review and editing. BvM, JD, JO: methodology, supervision, writing—review and editing.

Handling co-editors: Cecilia Rogmark and Philippe Wagner

*Acta* thanks Max Gordon and Christian Thomas Pollmann for help with peer review of this manuscript.
