# Supplementary material for: Machine learning-based prediction of short- and long-term mortality for shared decision-making in older hip fracture patients: the Dutch Hip Fracture Audit algorithms in 74,396 cases
Source: Acta Orthop. 2025 Jul 7;96:521–8. doi: 10.2340/17453674.2025.44248 (PMC12231630; doi:10.2340/17453674.2025.44248)
Supplement: Supplementary file 1 [file ActaO-96-44248-s1.pdf]

Appendices of

Machine Learning-Based Prediction of Mortality for Shared Decision-Making in Elderly Hip Fracture Patients: The Dutch Hip Fracture Algorithms in 74,396 Cases

Supplementary Table I. Technical Description of handling missing data, model development and statistical analysis

Handling missing data
In the data pre-processing stage, variables with more than 30% missing data were excluded.[1] The remaining missing data were imputed using the missForest algorithm.[1,2] This algorithm employs a non-parametric approach, effectively handling mix-type data (e.g. continuous and categorical data) based on averaging regression trees. It demonstrates computational efficiency and is particularly suited for high-dimensional data. In addition, it has minimal assumptions about structural aspects of data.[2] None of the selected variables for this study had more than 30% missing. We imputed missing values for gender (n = 104, 0.14%), ASA class (n = 5,576, 7.5%), prefracture living situation (n = 9,891, 13.3%), prefracture functional status (n = 4,814, 6.5%), dementia (10,180, 13.7%), daily living dependency (n = 3,341, 4.5%), risk of malnutrition (n = 5,237, 7.0%), osteoporosis (n = 11,102, 14.9%), fracture side (n = 146, 0.20%), fracture type (n = 3,171, 4.3%).
**Model development**
First, the total dataset was divided into a training-set (80%) and a hold-out test-set (20%), with stratification based on outcomes at 30-day, 90-day, and 1-year mortality intervals, for each outcome separately. While the same patient cases were considered for all three outcomes, we created cohorts for each outcome (thus for 30-day, 90-day and 1-year mortality a separate dataset), as these were not identical due to differences in available follow-up data for some patients. Second, variables determined from the feature selection step were used to train and internally validate several ML algorithms to choose the best-performing algorithm: Extreme Gradient Boosting (XGBoost), Random Forrest, Linear Support Vector Machine (SVM), Elastic-Net Penalized Logistic Regression, K-Nearest Neighbors and Logistic Regression (for explanations of each algorithm see Supplementary Appendix **Table II**). These algorithms were chosen based on prior ML studies and their binary classification capabilities.[3,4,5] The algorithms are supervised forms of ML, meaning that model development relies on the training of the algorithm with labelled data (the presence or absence of 30-day, 90-day and 1-year mortality). The rationale behind training multiple algorithms stems from the inherent unpredictability regarding which algorithm will exhibit superior performance, as the effectiveness of a particular algorithm can vary significantly depending on the dataset's characteristics and the complexity of the underlying patterns. Algorithm training was performed with 5-fold cross-validation in the training-set during grid search.[6,7] Cross-validation entails dividing data into a selected number of groups, named folds. First, the data are divided into 5 equally sized folds. Then, the algorithms were trained on 4 of the 5 folds (80% of the training data) and tested on the remaining fold (20% of the training data) using the performance metrics outlined in the following section. Grid search systematically evaluates a ML model's performance across a predefined set of parameter values, aiming to identify the optimal configuration—in this case, the one yielding the highest C-statistic. The parameters for the grid search of each ML algorithm can be found in the Supplementary Appendix **Table III**. Performance assessment for both training- and test-set was conducted using 1,000 bootstrap resamples of both sets, calculating performance metrics for each resample and computing point estimates and 95% confidence intervals (CI) for each metric. Subsequently, to address the class imbalance in our datasets, we used multiple classification thresholds: the default value of 0.50 and three custom thresholds based on the proportion of mortality cases in each dataset. We then empirically evaluated and compared the predictive performance of each model on the testing set using all four decision thresholds (0.10, 0.15, 0.25, and 0.50). Lastly, feature importance for the best performing algorithm across all outcomes was explored and illustrated in a bar chart. Feature importance refers to techniques used to assign a score to input features (predictive variables) based on their impact on the output of the ML-based probability calculator. It gives insight in the contribution of each feature to the output (i.e. explainable artificial intelligence).

Supplementary Table II. Short description of included algorithms

Machine learning algorithm	Short description

XGBoost algorithm	XGBoost, short for Extreme Gradient Boosting, is a powerful machine learning algorithm that implements gradient boosting. It builds an ensemble of decision trees sequentially, where each new tree attempts to correct the errors of the previous ones by optimizing a specific loss function. The algorithm is known for its scalability, speed, and performance, handling sparse data well and incorporating regularization to prevent overfitting. Unlike a random forest, which constructs a multitude of decision trees independently and aggregates their predictions, XGBoost builds trees in a sequential manner, with each tree focusing on the residuals of the previous ones to improve accuracy iteratively.
Random Forest	Random Forest is an ensemble learning algorithm that constructs multiple decision trees during training and merges their outputs to improve accuracy and control overfitting. Each tree in the forest is built using a random subset of the training data and features, ensuring diversity among the trees. The final prediction is made by aggregating the predictions from all the individual trees, typically through majority voting for classification or averaging for regression. This method enhances robustness and generalization compared to single decision trees, making it a popular choice for various machine learning tasks.
Linear Support vector machine	A Linear Support Vector Machine is a classification algorithm that distinguishes between two different outcomes by plotting data points in a multidimensional space and finding the optimal decision boundary. First, the data is transformed and represented as points in this space. The algorithm then identifies a hyperplane that maximizes the margin, which is the distance between the hyperplane and the nearest data points from each class, ensuring the best separation between the classes. Linear SVMs are particularly effective for linearly separable data (e.g the kernel is linear) and tend to perform well on large datasets due to their computational efficiency.
Elastic net Penalized logistic regression	Elastic Net Penalized Logistic Regression is a variant of logistic regression that fits an S-shaped probability curve to numerical data, using the log odds to predict binary outcomes. In this approach, penalties are applied to the model to prevent overfitting and manage multicollinearity, combining both L1 (lasso) and L2 (ridge) regularization techniques. These penalties shrink the coefficients of less important variables, effectively reducing the number of variables included in the model. This results in a more robust and interpretable model, particularly useful when dealing with high-dimensional data.
K-Nearest Neighbours	K-Nearest Neighbors (KNN) is a machine learning algorithm predicts based on the proximity of data points. It identifies the 'k' nearest neighbors of a given input in the feature space and assigns the most common class (for classification) or the average value (for regression) among them. KNN is a non-parametric, ‘lazy’ learning algorithm, meaning it doesn’t require a training phase and makes predictions directly based on the input data.
Logistic regression	Logistic regression is a statistical algorithm primarily used for binary classification tasks, where the goal is to predict one of two possible outcomes. It models the probability of a given input belonging to a particular class using a logistic function, which produces an S-shaped curve. This function transforms the linear combination of input features into a probability value between 0 and 1, which can then be used to classify the input. Logistic regression is widely used due to its simplicity, interpretability, and effectiveness in situations where the relationship between the features and the target is approximately linear.

Supplementary Table III. Hyperparameter tuning explained

Machine Learning Algorithm	Data scaling method	Hyperparameter tuning
Logistic regression	none	param_grid = {<br>'solver': ['lbfgs', 'saga','liblinear'],<br>'C': [0.01, 0.1, 1, 10, 100],<br>'class_weight': [None, 'balanced']<br>}
Elastic-net Penalized logistic regression	StandardScaler	param_grid = {<br>'sgdclassifier__alpha': [1e-4, 1e-3, 1e-2, 1e-1],<br>'sgdclassifier__l1_ratio': [0.0, 0.15, 0.5, 0.85, 1.0],<br>'sgdclassifier__max_iter': [1000, 2000, 3000],<br>'sgdclassifier__tol': [1e-3, 1e-4, 1e-5]<br>}
Linear Support Vector Machine	StandardScaler	param_grid = {<br>'linearsvc__C': [0.001, 0.01, 0.1, 1, 10],<br>'linearsvc__loss': ['squared_hinge', 'hinge'],<br>'linearsvc__max_iter': [1000, 5000, 10000],<br>'linearsvc__tol': [1e-4, 1e-5]<br>}
K Nearest Neighbours	None	param_grid = {'n_neighbors': np.arange(1, 100)}
Random Forest	None	param_grid = {<br>'n_estimators': [100, 200, 300, 1000],<br>'max_features': ['log2', 'sqrt'],<br>'max_depth': [None, 5, 8, 20],<br>'criterion': ['gini', 'entropy'],<br>'min_samples_split': [2, 6, 10],<br>'min_samples_leaf': [1, 2, 4],<br>}
XGBoost	None	param_grid = {<br>'n_estimators': [50, 100, 200],<br>'max_depth': [3, 4, 5, 6],<br>'learning_rate': [0.01, 0.05, 0.1],<br>'subsample': [0.6, 0.8, 1.0],<br>'colsample_bytree': [0.6, 0.8, 1.0]<br>}

Supplementary Table IV. Model performance metrics explained

C-statistic	Discrimination was assessed with the c-statistic (i.e. the area under the curve (AUC) of a receiver operating characteristic curve (ROC-curve)). The ROC-curve plots the sensitivity (true positive rate) against 1 - specificity (false positive rate). The c-statistic ranges from 0.50 to 1.0 with 1.0 indicating perfect discrimination (the model is always correct) and 0.50 indicating the random discrimination (the model's performance is akin to a coin toss). This differentiates between patients who had the outcome of interest (i.e. 30-day, 90-day and 1-year mortality) from those who had not.[8]
Calibration	Calibration was assessed with the calibration slope and calibration intercept of a calibration curve.[9] Calibration reflects the agreement between the observed outcome and the predicted outcome. It can be assessed and visualized by plotting the predicted probability (x-axis) versus the actual probability (y-axis) creating a calibration curve. The intercept indicates that predictions are systematically too high (intercept <0) or too low (intercept >0). The slope of the calibration curve reflects whether predictions were too extreme (low predictions too low and high predictions too high; a slope smaller than 1) or not extreme enough (low predictions not low enough and high predictions not high enough; a slope larger than 1). A perfect calibration curve has a slope of 1 and an intercept of 0.[9,10]
Sensitivity (recall)	Sensitivity, also known as the true positive rate or recall, corresponds to the proportion of positive observations that are correctly classified as positive compared with all predictions (TP / (FN+TP)). Sensitivity is 100% if all positive observations are classified as positive.
Specificity	Specificity, also known as the true negative rate, corresponds to the proportion of negative observations that are correctly classified as negative compared to all prediction (TN / (TN+FP)). Specificity is 100% if all negative observations are classified as negative.
PR-AUC	The PR-AUC (Precision-Recall Area Under the Curve) quantifies a model's performance by plotting precision (positive predictive value) against recall (sensitivity) across different thresholds. It summarizes the trade-off between precision and recall, with a higher PR-AUC indicating better performance in distinguishing between positive and negative cases, especially in imbalanced datasets.
Brier-score	The Brier score measures overall algorithm performance (composite of discrimination and calibration) by calculating the squared differences between actual outcomes and predictions, with a range from 0 (perfect model) to 1 (worst possible). The score also accounts for the incidence of outcomes in the dataset, with the upper limit of the Brier score being adjusted accordingly.
F1-score	F1-score tries to find a balance between precision and recall, and ranges from 0 being the lowest to 1 being the highest

AUC = Area under the curve; ROC = receiver operating curve; PRC = precision recall curve; TP = true positive; TN = true negative; FP = false positive; FN = false negative.

Supplementary Table V. Performance of Machine Learning algorithms in predicting 30-day, 90-day and 1-year mortality in the training-set after 1000 bootstrapping iterations using different thresholds for each outcome: 0.10 (30-day mortality), 0.15 (90-day mortality and 0.25 (1-year mortality).

**Algorithm**	XGBoost	Random Forest	Linear Support Vector Machine	Elastic-Net Penalized Logistic Regression	KNN	Logistic Regression*
**Metric (30-day mortality)**						
c-statistic	0.84 (0.83 - 0.84)	0.84 (0.83 - 0.84)	0.82 (0.81 - 0.82)	0.81 (0.80 - 0.81)	0.84 (0.83 - 0.84)	0.82 (0.81 - 0.82)
Calibration slope	1.12 (1.09 - 1.15)	1.24 (1.21 - 1.27)	0.95 (0.92 - 0.98)	1.07 (1.04 - 1.09)	1.09 (1.06 - 1.12)	0.99 (0.95 - 1.02)
Calibration intercept	-0.01 (-0.01 - -0.01)	-0.02 (-0.02 - -0.02)	0.00 (0.00 - 0.01)	-0.01 (-0.01 - -0.01)	0.00 (-0.01 - 0.00)	0.00 (-0.01 - 0.00)
Sensitivity	0.78 (0.77 - 0.79)	0.80 (0.79 - 0.81)	0.73 (0.72 - 0.75)	0.87 (0.86 - 0.88)	0.79 (0.78 - 0.80)	0.76 (0.75 - 0.78)
Specificity	0.74 (0.73 - 0.74)	0.72 (0.72 - 0.72)	0.74 (0.74 - 0.75)	0.56 (0.56 - 0.57)	0.73 (0.73 - 0.73)	0.72 (0.71 - 0.72)
PR-AUC	0.37 (0.36 - 0.39)	0.38 (0.36 - 0.39)	0.30 (0.29 - 0.31)	0.41 (0.40 - 0.42)	0.32 (0.31 - 0.34)	0.30 (0.29 - 0.32)
**Metric (90-day mortality)**						
c-statistic	0.83 (0.83 - 0.84)	0.82 (0.82 - 0.83)	0.81 (0.80 - 0.81)	0.81 (0.80 - 0.81)	0.82 (0.81 - 0.82)	0.81 (0.80 - 0.81)
Calibration slope	1.07 (1.05 - 1.09)	1.20 (1.18 - 1.23)	0.96 (0.94 - 0.99)	1.07 (1.04 - 1.09)	1.09 (1.07 - 1.12)	0.99 (0.96 - 1.01)
Calibration intercept	-0.01 (-0.01 - -0.01)	-0.03 (-0.03 - -0.03)	0.01 (0.00 - 0.01)	-0.01 (-0.01 - -0.01)	-0.01 (-0.01 - 0.00)	0.00 (0.00 - 0.00)
Sensitivity	0.79 (0.78 - 0.80)	0.80 (0.79 - 0.81)	0.74 (0.73 - 0.75)	0.75 (0.74 - 0.76)	0.78 (0.77 - 0.79)	0.87 (0.86 - 0.88)
Specificity	0.71 (0.70 - 0.71)	0.68 (0.68 - 0.69)	0.72 (0.72 - 0.72)	0.71 (0.71 - 0.71)	0.70 (0.70 - 0.70)	0.56 (0.56 - 0.57)
PR-AUC	0.48 (0.47 - 0.50)	0.47 (0.45 - 0.48)	0.41 (0.40 - 0.42)	0.41 (0.40 - 0.42)	0.43 (0.42 - 0.44)	0.41 (0.40 - 0.42)
**Metric (1-year mortality)**						
c-statistic	0.82 (0.81 - 0.82)	0.82 (0.81 - 0.82)	0.80 (0.79 - 0.80)	0.81 (0.80 - 0.81)	0.81 (0.80 - 0.81)	0.80 (0.79 - 0.80)
Calibration slope	1.03 (1.02 - 1.05)	1.16 (1.14 - 1.17)	0.98 (0.97 - 1.00)	1.07 (1.04 - 1.09)	1.07 (1.05 - 1.09)	1.00 (0.98 - 1.01)
Calibration intercept	-0.01 (-0.01 - 0.00)	-0.04 (-0.04 - -0.04)	0.00 (0.00 - 0.01)	-0.01 (-0.01 - -0.01)	-0.01 (-0.01 - 0.00)	0.00 (-0.01 - 0.00)
Sensitivity	0.78 (0.78 - 0.79)	0.79 (0.79 - 0.80)	0.74 (0.74 - 0.75)	0.87 (0.86 - 0.87)	0.78 (0.77 - 0.79)	0.75 (0.75 - 0.76)
Specificity	0.69 (0.69 - 0.70)	0.68 (0.67 - 0.68)	0.70 (0.70 - 0.71)	0.40 (0.39 - 0.41)	0.69 (0.68 - 0.69)	0.69 (0.69 - 0.69)
PR-AUC	0.61 (0.60 - 0.62)	0.61 (0.61 - 0.62)	0.57 (0.56 - 0.58)	0.41 (0.40 - 0.42)	0.58 (0.57 - 0.59)	0.57 (0.56 - 0.58)

XGBoost = Extreme Gradient Boosting
KNN = K-nearest neighbors
*The logistic regression was the final best performing algorithm for 30-day, 90-day and 1-year mortality prediction in the training-set.
PR-AUC = Area Under Precision Recall Curve.

Supplementary Table VI. An overview of all performance metrics for 30-day mortality with different thresholds (XGBoost)

XGBoost 30-day mortality								
Metric, threshold and data	Threshold 0.1 Train Data	Threshold 0.1 Test Data	Threshold 0.15 Train Data	Threshold 0.15 Test Data	Threshold 0.25 Train Data	Threshold 0.25 Test Data	Threshold 0.5 Train Data	Threshold 0.5 Test Data
f1	0.36 (0.35 - 0.36)	0.34 (0.32 - 0.35)	0.39 (0.38 - 0.40)	0.37 (0.35 - 0.39)	0.40 (0.39 - 0.41)	0.38 (0.36 - 0.41)	0.10 (0.09 - 0.11)	0.09 (0.07 - 0.11)
sensitivity	0.78 (0.77 - 0.79)	0.77 (0.75 - 0.79)	0.65 (0.64 - 0.67)	0.63 (0.60 - 0.66)	0.40 (0.39 - 0.41)	0.40 (0.37 - 0.42)	0.05 (0.05 - 0.06)	0.05 (0.04 - 0.06)
specificity	0.74 (0.73 - 0.74)	0.73 (0.72 - 0.74)	0.83 (0.83 - 0.83)	0.83 (0.82 - 0.83)	0.94 (0.94 - 0.94)	0.93 (0.93 - 0.94)	1.00 (1.00 - 1.00)	1.00 (0.99 - 1.00)
precision	0.23 (0.22 - 0.24)	0.22 (0.21 - 0.23)	0.28 (0.27 - 0.29)	0.26 (0.25 - 0.28)	0.39 (0.38 - 0.41)	0.37 (0.35 - 0.40)	0.69 (0.65 - 0.73)	0.54 (0.46 - 0.62)
c-statistic	0.84 (0.83 - 0.84)	0.83 (0.82 - 0.84)	0.84 (0.83 - 0.84)	0.83 (0.82 - 0.84)	0.84 (0.83 - 0.84)	0.83 (0.82 - 0.84)	0.84 (0.83 - 0.84)	0.83 (0.82 - 0.84)
pr_auc	0.37 (0.36 - 0.39)	0.34 (0.31 - 0.36)	0.37 (0.36 - 0.39)	0.34 (0.31 - 0.36)	0.37 (0.36 - 0.39)	0.34 (0.31 - 0.36)	0.37 (0.36 - 0.39)	0.34 (0.31 - 0.36)
brier	0.07 (0.07 - 0.07)	0.07 (0.07 - 0.07)	0.07 (0.07 - 0.07)	0.07 (0.07 - 0.07)	0.07 (0.07 - 0.07)	0.07 (0.07 - 0.07)	0.07 (0.07 - 0.07)	0.07 (0.07 - 0.07)
calibration_slope	1.12 (1.09 - 1.15)	1.03 (0.97 - 1.09)	1.12 (1.09 - 1.15)	1.03 (0.97 - 1.09)	1.12 (1.09 - 1.15)	1.03 (0.97 - 1.09)	1.12 (1.09 - 1.15)	1.03 (0.97 - 1.09)
calibration_intercept	-0.01 (-0.01 - -0.01)	-0.01 (-0.01 - -0.00)	-0.01 (-0.01 - -0.01)	-0.01 (-0.01 - -0.00)	-0.01 (-0.01 - -0.01)	-0.01 (-0.01 - -0.00)	-0.01 (-0.01 - -0.01)	-0.01 (-0.01 - -0.00)

Supplementary Table VII. An overview of all performance metrics for 90-day mortality with different thresholds (XGBoost)

XGBoost 90-day mortality								
Metric	Threshold 0.1 Train Data	Threshold 0.1 Test Data	Threshold 0.15 Train Data	Threshold 0.15 Test Data	Threshold 0.25 Train Data	Threshold 0.25 Test Data	Threshold 0.5 Train Data	Threshold 0.5 Test Data
f1	0.42 (0.41 - 0.42)	0.41 (0.40 - 0.42)	0.46 (0.45 - 0.46)	0.44 (0.43 - 0.45)	0.49 (0.48 - 0.50)	0.47 (0.46 - 0.49)	0.29 (0.27 - 0.30)	0.26 (0.24 - 0.28)
sensitivity	0.88 (0.87 - 0.88)	0.87 (0.86 - 0.89)	0.79 (0.78 - 0.80)	0.78 (0.76 - 0.79)	0.60 (0.58 - 0.61)	0.59 (0.57 - 0.61)	0.18 (0.17 - 0.19)	0.17 (0.15 - 0.18)

specificity	0.60 (0.59 - 0.60)	0.58 (0.57 - 0.59)	0.71 (0.70 - 0.71)	0.69 (0.69 - 0.70)	0.86 (0.85 - 0.86)	0.84 (0.84 - 0.85)	0.98 (0.98 - 0.99)	0.98 (0.98 - 0.98)
precision	0.27 (0.27 - 0.28)	0.27 (0.26 - 0.28)	0.32 (0.31 - 0.33)	0.31 (0.30 - 0.32)	0.42 (0.41 - 0.42)	0.40 (0.38 - 0.41)	0.66 (0.64 - 0.68)	0.60 (0.56 - 0.64)
c-statistic	0.83 (0.83 - 0.84)	0.81 (0.81 - 0.82)	0.83 (0.83 - 0.84)	0.81 (0.81 - 0.82)	0.83 (0.83 - 0.84)	0.81 (0.81 - 0.82)	0.83 (0.83 - 0.84)	0.81 (0.81 - 0.82)
pr_auc	0.48 (0.47 - 0.50)	0.44 (0.42 - 0.46)	0.48 (0.47 - 0.50)	0.44 (0.42 - 0.46)	0.48 (0.47 - 0.50)	0.44 (0.42 - 0.46)	0.48 (0.47 - 0.50)	0.44 (0.42 - 0.46)
brier	0.10 (0.10 - 0.10)	0.10 (0.10 - 0.11)	0.10 (0.10 - 0.10)	0.10 (0.10 - 0.11)	0.10 (0.10 - 0.10)	0.10 (0.10 - 0.11)	0.10 (0.10 - 0.10)	0.10 (0.10 - 0.11)
calibration_slope	1.07 (1.05 - 1.09)	0.98 (0.94 - 1.03)	1.07 (1.05 - 1.09)	0.98 (0.94 - 1.03)	1.07 (1.05 - 1.09)	0.98 (0.94 - 1.03)	1.07 (1.05 - 1.09)	0.98 (0.94 - 1.03)
calibration_intercept	-0.01 (-0.01 - -0.01)	0.00 (-0.00 - 0.01)	-0.01 (-0.01 - -0.01)	0.00 (-0.00 - 0.01)	-0.01 (-0.01 - -0.01)	0.00 (-0.00 - 0.01)	-0.01 (-0.01 - -0.01)	0.00 (-0.00 - 0.01)

Supplementary Table VIII. An overview of all performance metrics for 1-year mortality with different thresholds (XGBoost)

XGBoost 1-year mortality								
Metric	Threshold 0.1 Train Data	Threshold 0.1 Test Data	Threshold 0.15 Train Data	Threshold 0.15 Test Data	Threshold 0.25 Train Data	Threshold 0.25 Test Data	Threshold 0.5 Train Data	Threshold 0.5 Test Data
f1	0.51 (0.51 - 0.52)	0.51 (0.50 - 0.52)	0.55 (0.54 - 0.56)	0.54 (0.53 - 0.55)	0.59 (0.58 - 0.59)	0.57 (0.56 - 0.59)	0.50 (0.49 - 0.51)	0.47 (0.46 - 0.49)
sensitivity	0.95 (0.95 - 0.95)	0.95 (0.94 - 0.96)	0.90 (0.89 - 0.90)	0.89 (0.88 - 0.90)	0.78 (0.78 - 0.79)	0.77 (0.75 - 0.78)	0.40 (0.39 - 0.41)	0.38 (0.37 - 0.40)
specificity	0.39 (0.38 - 0.39)	0.38 (0.37 - 0.39)	0.52 (0.52 - 0.53)	0.52 (0.51 - 0.53)	0.69 (0.69 - 0.70)	0.68 (0.68 - 0.69)	0.93 (0.92 - 0.93)	0.92 (0.92 - 0.93)
precision	0.35 (0.35 - 0.36)	0.35 (0.34 - 0.36)	0.40 (0.39 - 0.40)	0.39 (0.38 - 0.40)	0.47 (0.46 - 0.48)	0.46 (0.44 - 0.47)	0.66 (0.65 - 0.67)	0.63 (0.61 - 0.65)
c-statistic	0.82 (0.81 - 0.82)	0.81 (0.80 - 0.81)	0.82 (0.81 - 0.82)	0.81 (0.80 - 0.81)	0.82 (0.81 - 0.82)	0.81 (0.80 - 0.81)	0.82 (0.81 - 0.82)	0.81 (0.80 - 0.81)
pr_auc	0.61 (0.60 - 0.62)	0.58 (0.57 - 0.60)	0.61 (0.60 - 0.62)	0.58 (0.57 - 0.60)	0.61 (0.60 - 0.62)	0.58 (0.57 - 0.60)	0.61 (0.60 - 0.62)	0.58 (0.57 - 0.60)
brier	0.14 (0.14 - 0.15)	0.15 (0.14 - 0.15)	0.14 (0.14 - 0.15)	0.15 (0.14 - 0.15)	0.14 (0.14 - 0.15)	0.15 (0.14 - 0.15)	0.14 (0.14 - 0.15)	0.15 (0.14 - 0.15)
calibration_slope	1.03 (1.02 - 1.05)	0.99 (0.96 - 1.02)	1.03 (1.02 - 1.05)	0.99 (0.96 - 1.02)	1.03 (1.02 - 1.05)	0.99 (0.96 - 1.02)	1.03 (1.02 - 1.05)	0.99 (0.96 - 1.02)

calibration_intercept	-0.01 (-0.01 - -0.00)	0.00 (-0.01 - 0.01)	-0.01 (-0.01 - -0.00)	0.00 (-0.01 - 0.01)	-0.01 (-0.01 - -0.00)	0.00 (-0.01 - 0.01)	-0.01 (-0.01 - -0.00)	0.00 (-0.01 - 0.01)

Supplementary Table IX. An overview of all performance metrics for 30-day mortality with different thresholds (Random Forest)

Random Forest 30-day mortality								
Metric	Threshold 0.1 Train Data	Threshold 0.1 Test Data	Threshold 0.15 Train Data	Threshold 0.15 Test Data	Threshold 0.25 Train Data	Threshold 0.25 Test Data	Threshold 0.5 Train Data	Threshold 0.5 Test Data
f1	0.35 (0.34 - 0.35)	0.33 (0.32 - 0.35)	0.39 (0.38 - 0.40)	0.37 (0.35 - 0.39)	0.39 (0.38 - 0.40)	0.37 (0.34 - 0.39)	0.05 (0.04 - 0.06)	0.05 (0.03 - 0.06)
sensitivity	0.80 (0.79 - 0.81)	0.78 (0.76 - 0.81)	0.66 (0.65 - 0.67)	0.64 (0.62 - 0.67)	0.36 (0.35 - 0.37)	0.35 (0.32 - 0.37)	0.02 (0.02 - 0.03)	0.03 (0.02 - 0.03)
specificity	0.72 (0.72 - 0.72)	0.72 (0.71 - 0.72)	0.83 (0.83 - 0.83)	0.82 (0.81 - 0.83)	0.95 (0.95 - 0.95)	0.95 (0.94 - 0.95)	1.00 (1.00 - 1.00)	1.00 (1.00 - 1.00)
precision	0.22 (0.22 - 0.23)	0.21 (0.20 - 0.22)	0.28 (0.27 - 0.29)	0.26 (0.24 - 0.27)	0.42 (0.41 - 0.44)	0.39 (0.36 - 0.42)	0.74 (0.68 - 0.80)	0.63 (0.50 - 0.76)
c-statistic	0.84 (0.83 - 0.84)	0.83 (0.82 - 0.84)	0.84 (0.83 - 0.84)	0.83 (0.82 - 0.84)	0.84 (0.83 - 0.84)	0.83 (0.82 - 0.84)	0.84 (0.83 - 0.84)	0.83 (0.82 - 0.84)
pr_auc	0.38 (0.36 - 0.39)	0.33 (0.31 - 0.36)	0.38 (0.36 - 0.39)	0.33 (0.31 - 0.36)	0.38 (0.36 - 0.39)	0.33 (0.31 - 0.36)	0.38 (0.36 - 0.39)	0.33 (0.31 - 0.36)
brier	0.07 (0.07 - 0.07)	0.07 (0.07 - 0.07)	0.07 (0.07 - 0.07)	0.07 (0.07 - 0.07)	0.07 (0.07 - 0.07)	0.07 (0.07 - 0.07)	0.07 (0.07 - 0.07)	0.07 (0.07 - 0.07)
calibration_slope	1.24 (1.21 - 1.27)	1.12 (1.06 - 1.19)	1.24 (1.21 - 1.27)	1.12 (1.06 - 1.19)	1.24 (1.21 - 1.27)	1.12 (1.06 - 1.19)	1.24 (1.21 - 1.27)	1.12 (1.06 - 1.19)
calibration_intercept	-0.02 (-0.02 - -0.02)	-0.02 (-0.02 - -0.01)	-0.02 (-0.02 - -0.02)	-0.02 (-0.02 - -0.01)	-0.02 (-0.02 - -0.02)	-0.02 (-0.02 - -0.01)	-0.02 (-0.02 - -0.02)	-0.02 (-0.02 - -0.01)

Supplementary Table X. An overview of all performance metrics for 90-day mortality with different thresholds (Random Forest)

Random Forest 90-day mortality								
Metric	Threshold 0.1 Train Data	Threshold 0.1 Test Data	Threshold 0.15 Train Data	Threshold 0.15 Test Data	Threshold 0.25 Train Data	Threshold 0.25 Test Data	Threshold 0.5 Train Data	Threshold 0.5 Test Data
f1	0.40 (0.39 - 0.40)	0.40 (0.38 - 0.41)	0.44 (0.44 - 0.45)	0.44 (0.42 - 0.45)	0.48 (0.47 - 0.49)	0.47 (0.46 - 0.49)	0.14 (0.13 - 0.15)	0.12 (0.11 - 0.14)
sensitivity	0.90 (0.90 - 0.91)	0.90 (0.89 - 0.91)	0.80 (0.79 - 0.81)	0.80 (0.79 - 0.82)	0.57 (0.56 - 0.59)	0.58 (0.56 - 0.60)	0.08 (0.07 - 0.08)	0.07 (0.06 - 0.08)

specificity	0.54 (0.54 - 0.55)	0.53 (0.52 - 0.54)	0.68 (0.68 - 0.69)	0.67 (0.66 - 0.68)	0.86 (0.85 - 0.86)	0.84 (0.84 - 0.85)	1.00 (0.99 - 1.00)	0.99 (0.99 - 0.99)
precision	0.26 (0.25 - 0.26)	0.25 (0.24 - 0.26)	0.31 (0.30 - 0.31)	0.30 (0.29 - 0.31)	0.41 (0.40 - 0.42)	0.40 (0.38 - 0.41)	0.75 (0.72 - 0.78)	0.64 (0.58 - 0.70)
c-statistic	0.82 (0.82 - 0.83)	0.81 (0.81 - 0.82)	0.82 (0.82 - 0.83)	0.81 (0.81 - 0.82)	0.82 (0.82 - 0.83)	0.81 (0.81 - 0.82)	0.82 (0.82 - 0.83)	0.81 (0.81 - 0.82)
pr_auc	0.47 (0.45 - 0.48)	0.44 (0.42 - 0.46)	0.47 (0.45 - 0.48)	0.44 (0.42 - 0.46)	0.47 (0.45 - 0.48)	0.44 (0.42 - 0.46)	0.47 (0.45 - 0.48)	0.44 (0.42 - 0.46)
brier	0.10 (0.10 - 0.10)	0.10 (0.10 - 0.11)	0.10 (0.10 - 0.10)	0.10 (0.10 - 0.11)	0.10 (0.10 - 0.10)	0.10 (0.10 - 0.11)	0.10 (0.10 - 0.10)	0.10 (0.10 - 0.11)
calibration_slope	1.20 (1.18 - 1.23)	1.14 (1.09 - 1.18)	1.20 (1.18 - 1.23)	1.14 (1.09 - 1.18)	1.20 (1.18 - 1.23)	1.14 (1.09 - 1.18)	1.20 (1.18 - 1.23)	1.14 (1.09 - 1.18)
calibration_intercept	-0.03 (-0.03 - -0.03)	-0.02 (-0.03 - -0.02)	-0.03 (-0.03 - -0.03)	-0.02 (-0.03 - -0.02)	-0.03 (-0.03 - -0.03)	-0.02 (-0.03 - -0.02)	-0.03 (-0.03 - -0.03)	-0.02 (-0.03 - -0.02)

Supplementary Table XI. An overview of all performance metrics for 1-year mortality with different thresholds (Random Forest)

Random Forest 1-year mortality								
Metric	Threshold 0.1 Train Data	Threshold 0.1 Test Data	Threshold 0.15 Train Data	Threshold 0.15 Test Data	Threshold 0.25 Train Data	Threshold 0.25 Test Data	Threshold 0.5 Train Data	Threshold 0.5 Test Data
f1	0.50 (0.49 - 0.50)	0.49 (0.48 - 0.50)	0.54 (0.53 - 0.54)	0.53 (0.52 - 0.54)	0.58 (0.58 - 0.59)	0.57 (0.56 - 0.58)	0.46 (0.45 - 0.47)	0.44 (0.42 - 0.46)
sensitivity	0.97 (0.96 - 0.97)	0.96 (0.96 - 0.97)	0.92 (0.91 - 0.92)	0.91 (0.91 - 0.92)	0.79 (0.79 - 0.80)	0.78 (0.77 - 0.79)	0.35 (0.34 - 0.36)	0.33 (0.32 - 0.35)
specificity	0.33 (0.32 - 0.33)	0.32 (0.31 - 0.33)	0.48 (0.47 - 0.48)	0.48 (0.47 - 0.49)	0.68 (0.67 - 0.68)	0.67 (0.66 - 0.68)	0.95 (0.94 - 0.95)	0.94 (0.93 - 0.94)
precision	0.33 (0.33 - 0.34)	0.33 (0.32 - 0.34)	0.38 (0.37 - 0.38)	0.38 (0.37 - 0.39)	0.46 (0.46 - 0.47)	0.45 (0.44 - 0.47)	0.69 (0.68 - 0.70)	0.65 (0.63 - 0.67)
c-statistic	0.82 (0.81 - 0.82)	0.80 (0.80 - 0.81)	0.82 (0.81 - 0.82)	0.80 (0.80 - 0.81)	0.82 (0.81 - 0.82)	0.80 (0.80 - 0.81)	0.82 (0.81 - 0.82)	0.80 (0.80 - 0.81)
pr_auc	0.61 (0.61 - 0.62)	0.58 (0.56 - 0.60)	0.61 (0.61 - 0.62)	0.58 (0.56 - 0.60)	0.61 (0.61 - 0.62)	0.58 (0.56 - 0.60)	0.61 (0.61 - 0.62)	0.58 (0.56 - 0.60)
brier	0.14 (0.14 - 0.15)	0.15 (0.14 - 0.15)	0.14 (0.14 - 0.15)	0.15 (0.14 - 0.15)	0.14 (0.14 - 0.15)	0.15 (0.14 - 0.15)	0.14 (0.14 - 0.15)	0.15 (0.14 - 0.15)
calibration_slope	1.16 (1.14 - 1.17)	1.10 (1.07 - 1.14)	1.16 (1.14 - 1.17)	1.10 (1.07 - 1.14)	1.16 (1.14 - 1.17)	1.10 (1.07 - 1.14)	1.16 (1.14 - 1.17)	1.10 (1.07 - 1.14)
calibration_intercept	-0.04 (-0.04 - -0.04)	-0.03 (-0.04 - -0.02)	-0.04 (-0.04 - -0.04)	-0.03 (-0.04 - -0.02)	-0.04 (-0.04 - -0.04)	-0.03 (-0.04 - -0.02)	-0.04 (-0.04 - -0.04)	-0.03 (-0.04 - -0.02)

Supplementary Table XII. An overview of all performance metrics for 30-day mortality with different thresholds (Linear Support Vector Machine)

Linear Support Vector Machine 30-day mortality								
Metric	Threshold 0.1 Train Data	Threshold 0.1 Test Data	Threshold 0.15 Train Data	Threshold 0.15 Test Data	Threshold 0.25 Train Data	Threshold 0.25 Test Data	Threshold 0.5 Train Data	Threshold 0.5 Test Data
f1	0.34 (0.33 - 0.35)	0.33 (0.32 - 0.35)	0.37 (0.36 - 0.38)	0.37 (0.35 - 0.38)	0.34 (0.33 - 0.35)	0.35 (0.33 - 0.37)	0.10 (0.09 - 0.11)	0.11 (0.09 - 0.14)
sensitivity	0.73 (0.72 - 0.75)	0.73 (0.71 - 0.76)	0.58 (0.57 - 0.59)	0.59 (0.57 - 0.62)	0.33 (0.32 - 0.34)	0.35 (0.33 - 0.38)	0.06 (0.05 - 0.06)	0.06 (0.05 - 0.08)
specificity	0.74 (0.74 - 0.75)	0.74 (0.74 - 0.75)	0.84 (0.84 - 0.85)	0.84 (0.83 - 0.85)	0.94 (0.93 - 0.94)	0.94 (0.93 - 0.94)	0.99 (0.99 - 0.99)	0.99 (0.99 - 0.99)
precision	0.22 (0.22 - 0.23)	0.22 (0.21 - 0.23)	0.27 (0.26 - 0.28)	0.26 (0.25 - 0.28)	0.34 (0.33 - 0.36)	0.35 (0.32 - 0.37)	0.47 (0.43 - 0.51)	0.49 (0.41 - 0.56)
c-statistic	0.82 (0.81 - 0.82)	0.82 (0.81 - 0.83)	0.82 (0.81 - 0.82)	0.82 (0.81 - 0.83)	0.82 (0.81 - 0.82)	0.82 (0.81 - 0.83)	0.82 (0.81 - 0.82)	0.82 (0.81 - 0.83)
pr_auc	0.30 (0.29 - 0.31)	0.30 (0.28 - 0.33)	0.30 (0.29 - 0.31)	0.30 (0.28 - 0.33)	0.30 (0.29 - 0.31)	0.30 (0.28 - 0.33)	0.30 (0.29 - 0.31)	0.30 (0.28 - 0.33)
brier	0.07 (0.07 - 0.07)	0.07 (0.07 - 0.07)	0.07 (0.07 - 0.07)	0.07 (0.07 - 0.07)	0.07 (0.07 - 0.07)	0.07 (0.07 - 0.07)	0.07 (0.07 - 0.07)	0.07 (0.07 - 0.07)
calibration_slope	0.95 (0.92 - 0.98)	0.93 (0.87 - 0.99)	0.95 (0.92 - 0.98)	0.93 (0.87 - 0.99)	0.95 (0.92 - 0.98)	0.93 (0.87 - 0.99)	0.95 (0.92 - 0.98)	0.93 (0.87 - 0.99)
calibration_intercept	0.00 (0.00 - 0.01)	0.00 (-0.00 - 0.01)	0.00 (0.00 - 0.01)	0.00 (-0.00 - 0.01)	0.00 (0.00 - 0.01)	0.00 (-0.00 - 0.01)	0.00 (0.00 - 0.01)	0.00 (-0.00 - 0.01)

Supplementary Table XIII. An overview of all performance metrics for 90-day mortality with different thresholds (Linear Support Vector Machine)

Linear Support Vector Machine 90-day mortality								
Metric	Threshold 0.1 Train Data	Threshold 0.1 Test Data	Threshold 0.15 Train Data	Threshold 0.15 Test Data	Threshold 0.25 Train Data	Threshold 0.25 Test Data	Threshold 0.5 Train Data	Threshold 0.5 Test Data
f1	0.40 (0.40 - 0.41)	0.41 (0.40 - 0.42)	0.44 (0.43 - 0.45)	0.44 (0.43 - 0.46)	0.45 (0.44 - 0.46)	0.46 (0.44 - 0.47)	0.24 (0.23 - 0.25)	0.24 (0.22 - 0.26)

sensitivity	0.86 (0.85 - 0.86)	0.87 (0.86 - 0.88)	0.74 (0.73 - 0.75)	0.76 (0.74 - 0.77)	0.52 (0.51 - 0.53)	0.55 (0.52 - 0.57)	0.15 (0.14 - 0.16)	0.16 (0.14 - 0.17)
specificity	0.59 (0.58 - 0.59)	0.58 (0.57 - 0.59)	0.72 (0.72 - 0.72)	0.71 (0.70 - 0.72)	0.86 (0.86 - 0.86)	0.85 (0.85 - 0.86)	0.98 (0.98 - 0.98)	0.98 (0.98 - 0.98)
precision	0.26 (0.26 - 0.27)	0.27 (0.26 - 0.28)	0.31 (0.31 - 0.32)	0.31 (0.30 - 0.33)	0.39 (0.38 - 0.40)	0.39 (0.38 - 0.41)	0.55 (0.53 - 0.57)	0.56 (0.52 - 0.59)
c-statistic	0.81 (0.80 - 0.81)	0.81 (0.80 - 0.82)	0.81 (0.80 - 0.81)	0.81 (0.80 - 0.82)	0.81 (0.80 - 0.81)	0.81 (0.80 - 0.82)	0.81 (0.80 - 0.81)	0.81 (0.80 - 0.82)
pr_auc	0.41 (0.40 - 0.42)	0.41 (0.39 - 0.44)	0.41 (0.40 - 0.42)	0.41 (0.39 - 0.44)	0.41 (0.40 - 0.42)	0.41 (0.39 - 0.44)	0.41 (0.40 - 0.42)	0.41 (0.39 - 0.44)
brier	0.11 (0.10 - 0.11)	0.11 (0.10 - 0.11)	0.11 (0.10 - 0.11)	0.11 (0.10 - 0.11)	0.11 (0.10 - 0.11)	0.11 (0.10 - 0.11)	0.11 (0.10 - 0.11)	0.11 (0.10 - 0.11)
calibration_slope	0.96 (0.94 - 0.99)	0.96 (0.92 - 1.01)	0.96 (0.94 - 0.99)	0.96 (0.92 - 1.01)	0.96 (0.94 - 0.99)	0.96 (0.92 - 1.01)	0.96 (0.94 - 0.99)	0.96 (0.92 - 1.01)
calibration_intercept	0.01 (0.00 - 0.01)	0.00 (-0.00 - 0.01)	0.01 (0.00 - 0.01)	0.00 (-0.00 - 0.01)	0.01 (0.00 - 0.01)	0.00 (-0.00 - 0.01)	0.01 (0.00 - 0.01)	0.00 (-0.00 - 0.01)

Supplementary Table XIV. An overview of all performance metrics for 1-year mortality with different thresholds (Linear Support Vector Machine)

Linear Support Vector Machine 1-year mortality								
Metric	Threshold 0.1 Train Data	Threshold 0.1 Test Data	Threshold 0.15 Train Data	Threshold 0.15 Test Data	Threshold 0.25 Train Data	Threshold 0.25 Test Data	Threshold 0.5 Train Data	Threshold 0.5 Test Data
f1	0.50 (0.50 - 0.51)	0.50 (0.49 - 0.51)	0.54 (0.54 - 0.55)	0.54 (0.53 - 0.55)	0.57 (0.57 - 0.58)	0.57 (0.56 - 0.59)	0.46 (0.45 - 0.47)	0.45 (0.44 - 0.47)
sensitivity	0.95 (0.95 - 0.96)	0.95 (0.94 - 0.96)	0.89 (0.89 - 0.90)	0.89 (0.88 - 0.90)	0.74 (0.74 - 0.75)	0.74 (0.73 - 0.76)	0.37 (0.36 - 0.37)	0.36 (0.35 - 0.38)
specificity	0.37 (0.36 - 0.37)	0.36 (0.35 - 0.37)	0.52 (0.51 - 0.52)	0.52 (0.51 - 0.53)	0.70 (0.70 - 0.71)	0.71 (0.70 - 0.72)	0.92 (0.92 - 0.92)	0.92 (0.92 - 0.93)
precision	0.34 (0.34 - 0.35)	0.34 (0.33 - 0.35)	0.39 (0.38 - 0.40)	0.39 (0.38 - 0.40)	0.47 (0.46 - 0.47)	0.47 (0.45 - 0.48)	0.62 (0.61 - 0.63)	0.61 (0.59 - 0.64)
c-statistic	0.80 (0.79 - 0.80)	0.80 (0.79 - 0.81)	0.80 (0.79 - 0.80)	0.80 (0.79 - 0.81)	0.80 (0.79 - 0.80)	0.80 (0.79 - 0.81)	0.80 (0.79 - 0.80)	0.80 (0.79 - 0.81)
pr_auc	0.57 (0.56 - 0.58)	0.56 (0.54 - 0.58)	0.57 (0.56 - 0.58)	0.56 (0.54 - 0.58)	0.57 (0.56 - 0.58)	0.56 (0.54 - 0.58)	0.57 (0.56 - 0.58)	0.56 (0.54 - 0.58)

brier	0.15 (0.15 - 0.15)	0.15 (0.15 - 0.15)	0.15 (0.15 - 0.15)	0.15 (0.15 - 0.15)	0.15 (0.15 - 0.15)	0.15 (0.15 - 0.15)	0.15 (0.15 - 0.15)	0.15 (0.15 - 0.15)
calibration_slope	0.98 (0.97 - 1.00)	0.98 (0.94 - 1.01)	0.98 (0.97 - 1.00)	0.98 (0.94 - 1.01)	0.98 (0.97 - 1.00)	0.98 (0.94 - 1.01)	0.98 (0.97 - 1.00)	0.98 (0.94 - 1.01)
calibration_intercept	0.00 (-0.00 - 0.01)	0.01 (-0.00 - 0.01)	0.00 (-0.00 - 0.01)	0.01 (-0.00 - 0.01)	0.00 (-0.00 - 0.01)	0.01 (-0.00 - 0.01)	0.00 (-0.00 - 0.01)	0.01 (-0.00 - 0.01)

Supplementary Table XV. An overview of all performance metrics for 30-day mortality with different thresholds (Elastic-Net Penalized Logistic Regression)

Elastic-Net Penalized Logistic Regression 30-day mortality								
Metric	Threshold 0.1 Train Data	Threshold 0.1 Test Data	Threshold 0.15 Train Data	Threshold 0.15 Test Data	Threshold 0.25 Train Data	Threshold 0.25 Test Data	Threshold 0.5 Train Data	Threshold 0.5 Test Data
f1	0.34 (0.33 - 0.34)	0.33 (0.31 - 0.34)	0.37 (0.36 - 0.38)	0.36 (0.34 - 0.38)	0.33 (0.32 - 0.34)	0.35 (0.32 - 0.37)	0.06 (0.05 - 0.07)	0.06 (0.04 - 0.07)
sensitivity	0.75 (0.74 - 0.76)	0.75 (0.73 - 0.78)	0.58 (0.57 - 0.60)	0.59 (0.56 - 0.62)	0.31 (0.30 - 0.32)	0.33 (0.30 - 0.36)	0.03 (0.03 - 0.04)	0.03 (0.02 - 0.04)
specificity	0.73 (0.73 - 0.73)	0.73 (0.72 - 0.73)	0.84 (0.84 - 0.84)	0.84 (0.83 - 0.84)	0.95 (0.94 - 0.95)	0.94 (0.94 - 0.95)	1.00 (1.00 - 1.00)	1.00 (1.00 - 1.00)
precision	0.22 (0.21 - 0.22)	0.21 (0.20 - 0.22)	0.27 (0.26 - 0.28)	0.26 (0.24 - 0.28)	0.36 (0.35 - 0.38)	0.36 (0.34 - 0.39)	0.49 (0.43 - 0.54)	0.45 (0.35 - 0.55)
c-statistic	0.82 (0.81 - 0.82)	0.82 (0.81 - 0.83)	0.82 (0.81 - 0.82)	0.82 (0.81 - 0.83)	0.82 (0.81 - 0.82)	0.82 (0.81 - 0.83)	0.82 (0.81 - 0.82)	0.82 (0.81 - 0.83)
pr_auc	0.30 (0.29 - 0.31)	0.30 (0.28 - 0.32)	0.30 (0.29 - 0.31)	0.30 (0.28 - 0.32)	0.30 (0.29 - 0.31)	0.30 (0.28 - 0.32)	0.30 (0.29 - 0.31)	0.30 (0.28 - 0.32)
brier	0.07 (0.07 - 0.07)	0.07 (0.07 - 0.07)	0.07 (0.07 - 0.07)	0.07 (0.07 - 0.07)	0.07 (0.07 - 0.07)	0.07 (0.07 - 0.07)	0.07 (0.07 - 0.07)	0.07 (0.07 - 0.07)
calibration_slope	1.06 (1.02 - 1.09)	1.04 (0.97 - 1.11)	1.06 (1.02 - 1.09)	1.04 (0.97 - 1.11)	1.06 (1.02 - 1.09)	1.04 (0.97 - 1.11)	1.06 (1.02 - 1.09)	1.04 (0.97 - 1.11)
calibration_intercept	-0.01 (-0.01 - -0.00)	-0.01 (-0.01 - -0.00)	-0.01 (-0.01 - -0.00)	-0.01 (-0.01 - -0.00)	-0.01 (-0.01 - -0.00)	-0.01 (-0.01 - -0.00)	-0.01 (-0.01 - -0.00)	-0.01 (-0.01 - -0.00)

Supplementary Table XVI. An overview of all performance metrics for 90-day mortality with different thresholds (Elastic-Net Penalized Logistic Regression)

Elastic-Net Penalized Logistic Regression 90-day mortality								
Metric	Threshold 0.1 Train Data	Threshold 0.1 Test Data	Threshold 0.15 Train Data	Threshold 0.15 Test Data	Threshold 0.25 Train Data	Threshold 0.25 Test Data	Threshold 0.5 Train Data	Threshold 0.5 Test Data
f1	0.40 (0.39 - 0.40)	0.40 (0.39 - 0.41)	0.44 (0.43 - 0.44)	0.44 (0.43 - 0.45)	0.44 (0.44 - 0.45)	0.46 (0.44 - 0.47)	0.18 (0.17 - 0.19)	0.18 (0.16 - 0.20)
sensitivity	0.87 (0.86 - 0.88)	0.89 (0.87 - 0.90)	0.75 (0.74 - 0.76)	0.77 (0.75 - 0.78)	0.51 (0.49 - 0.52)	0.53 (0.51 - 0.55)	0.11 (0.10 - 0.11)	0.11 (0.10 - 0.12)
specificity	0.56 (0.56 - 0.57)	0.55 (0.55 - 0.56)	0.71 (0.71 - 0.71)	0.70 (0.69 - 0.71)	0.87 (0.86 - 0.87)	0.86 (0.85 - 0.87)	0.99 (0.98 - 0.99)	0.99 (0.98 - 0.99)
precision	0.26 (0.25 - 0.26)	0.26 (0.25 - 0.27)	0.31 (0.30 - 0.32)	0.31 (0.30 - 0.32)	0.40 (0.39 - 0.41)	0.40 (0.38 - 0.42)	0.56 (0.54 - 0.59)	0.57 (0.52 - 0.61)
c-statistic	0.81 (0.80 - 0.81)	0.81 (0.80 - 0.82)	0.81 (0.80 - 0.81)	0.81 (0.80 - 0.82)	0.81 (0.80 - 0.81)	0.81 (0.80 - 0.82)	0.81 (0.80 - 0.81)	0.81 (0.80 - 0.82)
pr_auc	0.41 (0.40 - 0.42)	0.42 (0.39 - 0.44)	0.41 (0.40 - 0.42)	0.42 (0.39 - 0.44)	0.41 (0.40 - 0.42)	0.42 (0.39 - 0.44)	0.41 (0.40 - 0.42)	0.42 (0.39 - 0.44)
brier	0.11 (0.10 - 0.11)	0.11 (0.10 - 0.11)	0.11 (0.10 - 0.11)	0.11 (0.10 - 0.11)	0.11 (0.10 - 0.11)	0.11 (0.10 - 0.11)	0.11 (0.10 - 0.11)	0.11 (0.10 - 0.11)
calibration_slope	1.07 (1.04 - 1.09)	1.06 (1.02 - 1.11)	1.07 (1.04 - 1.09)	1.06 (1.02 - 1.11)	1.07 (1.04 - 1.09)	1.06 (1.02 - 1.11)	1.07 (1.04 - 1.09)	1.06 (1.02 - 1.11)
calibration_intercept	-0.01 (-0.01 - - 0.01)	-0.01 (-0.02 - - 0.00)	-0.01 (-0.01 - - 0.01)	-0.01 (-0.02 - - 0.00)	-0.01 (-0.01 - - 0.01)	-0.01 (-0.02 - - 0.00)	-0.01 (-0.01 - - 0.01)	-0.01 (-0.02 - -0.00)

Supplementary Table XVII. An overview of all performance metrics for 1-year mortality with different thresholds (Elastic-Net Penalized Logistic Regression)

Elastic-Net Penalized Logistic Regression 1-year-day mortality								
Metric	Threshold 0.1 Train Data	Threshold 0.1 Test Data	Threshold 0.15 Train Data	Threshold 0.15 Test Data	Threshold 0.25 Train Data	Threshold 0.25 Test Data	Threshold 0.5 Train Data	Threshold 0.5 Test Data
f1	0.50 (0.49 - 0.50)	0.49 (0.48 - 0.50)	0.54 (0.53 - 0.54)	0.53 (0.52 - 0.55)	0.57 (0.57 - 0.58)	0.57 (0.56 - 0.58)	0.45 (0.44 - 0.46)	0.45 (0.43 - 0.47)
sensitivity	0.96 (0.95 - 0.96)	0.96 (0.95 - 0.96)	0.90 (0.90 - 0.91)	0.90 (0.89 - 0.91)	0.75 (0.75 - 0.76)	0.75 (0.74 - 0.76)	0.35 (0.35 - 0.36)	0.35 (0.34 - 0.37)

specificity	0.35 (0.34 - 0.35)	0.34 (0.33 - 0.35)	0.49 (0.49 - 0.50)	0.50 (0.49 - 0.51)	0.69 (0.69 - 0.70)	0.70 (0.69 - 0.71)	0.93 (0.92 - 0.93)	0.93 (0.92 - 0.93)
precision	0.34 (0.33 - 0.34)	0.33 (0.32 - 0.34)	0.38 (0.38 - 0.39)	0.38 (0.37 - 0.39)	0.46 (0.45 - 0.47)	0.46 (0.45 - 0.48)	0.62 (0.61 - 0.63)	0.62 (0.60 - 0.64)
c-statistic	0.80 (0.79 - 0.80)	0.80 (0.79 - 0.81)	0.80 (0.79 - 0.80)	0.80 (0.79 - 0.81)	0.80 (0.79 - 0.80)	0.80 (0.79 - 0.81)	0.80 (0.79 - 0.80)	0.80 (0.79 - 0.81)
pr_auc	0.57 (0.56 - 0.58)	0.56 (0.54 - 0.58)	0.57 (0.56 - 0.58)	0.56 (0.54 - 0.58)	0.57 (0.56 - 0.58)	0.56 (0.54 - 0.58)	0.57 (0.56 - 0.58)	0.56 (0.54 - 0.58)
brier	0.15 (0.15 - 0.15)	0.15 (0.15 - 0.15)	0.15 (0.15 - 0.15)	0.15 (0.15 - 0.15)	0.15 (0.15 - 0.15)	0.15 (0.15 - 0.15)	0.15 (0.15 - 0.15)	0.15 (0.15 - 0.15)
calibration_slope	1.02 (1.00 - 1.04)	1.01 (0.98 - 1.05)	1.02 (1.00 - 1.04)	1.01 (0.98 - 1.05)	1.02 (1.00 - 1.04)	1.01 (0.98 - 1.05)	1.02 (1.00 - 1.04)	1.01 (0.98 - 1.05)
calibration_intercept	-0.01 (-0.01 - -0.00)	-0.00 (-0.01 - 0.00)	-0.01 (-0.01 - -0.00)	-0.00 (-0.01 - 0.00)	-0.01 (-0.01 - -0.00)	-0.00 (-0.01 - 0.00)	-0.01 (-0.01 - -0.00)	-0.00 (-0.01 - 0.00)

Supplementary Table XVIII. An overview of all performance metrics for 30-day mortality with different thresholds (K-nearest neighbours)

K-nearest neighbours 30-day mortality								
Metric	Threshold 0.1 Train Data	Threshold 0.1 Test Data	Threshold 0.15 Train Data	Threshold 0.15 Test Data	Threshold 0.25 Train Data	Threshold 0.25 Test Data	Threshold 0.5 Train Data	Threshold 0.5 Test Data
f1	0.35 (0.34 - 0.36)	0.33 (0.31 - 0.34)	0.38 (0.37 - 0.39)	0.37 (0.35 - 0.38)	0.37 (0.35 - 0.38)	0.37 (0.34 - 0.39)	0.05 (0.04 - 0.05)	0.05 (0.03 - 0.06)
sensitivity	0.79 (0.78 - 0.80)	0.76 (0.73 - 0.78)	0.63 (0.61 - 0.64)	0.62 (0.59 - 0.64)	0.37 (0.35 - 0.38)	0.38 (0.35 - 0.40)	0.02 (0.02 - 0.03)	0.03 (0.02 - 0.03)
specificity	0.73 (0.73 - 0.73)	0.73 (0.72 - 0.73)	0.84 (0.83 - 0.84)	0.83 (0.82 - 0.84)	0.94 (0.93 - 0.94)	0.93 (0.93 - 0.94)	1.00 (1.00 - 1.00)	1.00 (1.00 - 1.00)
precision	0.23 (0.22 - 0.23)	0.21 (0.20 - 0.22)	0.28 (0.27 - 0.29)	0.26 (0.25 - 0.28)	0.36 (0.35 - 0.38)	0.35 (0.33 - 0.38)	0.60 (0.54 - 0.67)	0.58 (0.45 - 0.71)
c-statistic	0.84 (0.83 - 0.84)	0.82 (0.81 - 0.83)	0.84 (0.83 - 0.84)	0.82 (0.81 - 0.83)	0.84 (0.83 - 0.84)	0.82 (0.81 - 0.83)	0.84 (0.83 - 0.84)	0.82 (0.81 - 0.83)
pr_auc	0.32 (0.31 - 0.34)	0.30 (0.28 - 0.33)	0.32 (0.31 - 0.34)	0.30 (0.28 - 0.33)	0.32 (0.31 - 0.34)	0.30 (0.28 - 0.33)	0.32 (0.31 - 0.34)	0.30 (0.28 - 0.33)
brier	0.07 (0.07 - 0.07)	0.07 (0.07 - 0.07)	0.07 (0.07 - 0.07)	0.07 (0.07 - 0.07)	0.07 (0.07 - 0.07)	0.07 (0.07 - 0.07)	0.07 (0.07 - 0.07)	0.07 (0.07 - 0.07)
calibration_slope	1.09 (1.06 - 1.12)	1.00 (0.94 - 1.06)	1.09 (1.06 - 1.12)	1.00 (0.94 - 1.06)	1.09 (1.06 - 1.12)	1.00 (0.94 - 1.06)	1.09 (1.06 - 1.12)	1.00 (0.94 - 1.06)

calibration_intercept	-0.00 (-0.01 - -0.00)	0.00 (-0.00 - 0.01)	-0.00 (-0.01 - -0.00)	0.00 (-0.00 - 0.01)	-0.00 (-0.01 - -0.00)	0.00 (-0.00 - 0.01)	-0.00 (-0.01 - -0.00)	0.00 (-0.00 - 0.01)

Supplementary Table XIX. An overview of all performance metrics for 90-day mortality with different thresholds (K-nearest neighbours)

K-nearest neighbours 90-day mortality								
Metric	Threshold 0.1 Train Data	Threshold 0.1 Test Data	Threshold 0.15 Train Data	Threshold 0.15 Test Data	Threshold 0.25 Train Data	Threshold 0.25 Test Data	Threshold 0.5 Train Data	Threshold 0.5 Test Data
f1	0.40 (0.40 - 0.41)	0.40 (0.39 - 0.42)	0.45 (0.44 - 0.45)	0.44 (0.43 - 0.45)	0.46 (0.45 - 0.47)	0.45 (0.44 - 0.47)	0.17 (0.16 - 0.18)	0.16 (0.14 - 0.18)
sensitivity	0.88 (0.88 - 0.89)	0.88 (0.87 - 0.90)	0.78 (0.77 - 0.79)	0.78 (0.76 - 0.80)	0.55 (0.54 - 0.56)	0.54 (0.52 - 0.56)	0.10 (0.09 - 0.10)	0.09 (0.08 - 0.10)
specificity	0.57 (0.56 - 0.57)	0.56 (0.55 - 0.57)	0.70 (0.70 - 0.70)	0.69 (0.68 - 0.70)	0.86 (0.86 - 0.86)	0.85 (0.84 - 0.85)	0.99 (0.99 - 0.99)	0.99 (0.99 - 0.99)
precision	0.26 (0.26 - 0.27)	0.26 (0.25 - 0.27)	0.31 (0.30 - 0.32)	0.31 (0.30 - 0.32)	0.40 (0.39 - 0.41)	0.39 (0.37 - 0.41)	0.63 (0.60 - 0.65)	0.58 (0.53 - 0.63)
c-statistic	0.82 (0.81 - 0.82)	0.80 (0.79 - 0.81)	0.82 (0.81 - 0.82)	0.80 (0.79 - 0.81)	0.82 (0.81 - 0.82)	0.80 (0.79 - 0.81)	0.82 (0.81 - 0.82)	0.80 (0.79 - 0.81)
pr_auc	0.43 (0.42 - 0.44)	0.40 (0.38 - 0.42)	0.43 (0.42 - 0.44)	0.40 (0.38 - 0.42)	0.43 (0.42 - 0.44)	0.40 (0.38 - 0.42)	0.43 (0.42 - 0.44)	0.40 (0.38 - 0.42)
brier	0.10 (0.10 - 0.10)	0.11 (0.10 - 0.11)	0.10 (0.10 - 0.10)	0.11 (0.10 - 0.11)	0.10 (0.10 - 0.10)	0.11 (0.10 - 0.11)	0.10 (0.10 - 0.10)	0.11 (0.10 - 0.11)
calibration_slope	1.09 (1.07 - 1.12)	1.04 (0.99 - 1.09)	1.09 (1.07 - 1.12)	1.04 (0.99 - 1.09)	1.09 (1.07 - 1.12)	1.04 (0.99 - 1.09)	1.09 (1.07 - 1.12)	1.04 (0.99 - 1.09)
calibration_intercept	-0.01 (-0.01 - -0.00)	0.00 (-0.00 - 0.01)	-0.01 (-0.01 - -0.00)	0.00 (-0.00 - 0.01)	-0.01 (-0.01 - -0.00)	0.00 (-0.00 - 0.01)	-0.01 (-0.01 - -0.00)	0.00 (-0.00 - 0.01)

Supplementary Table XX. An overview of all performance metrics for 1-year mortality with different thresholds (K-nearest neighbours)

K-nearest neighbours 1-year mortality								
Metric	Threshold 0.1 Train Data	Threshold 0.1 Test Data	Threshold 0.15 Train Data	Threshold 0.15 Test Data	Threshold 0.25 Train Data	Threshold 0.25 Test Data	Threshold 0.5 Train Data	Threshold 0.5 Test Data
f1	0.51 (0.51 - 0.52)	0.51 (0.49 - 0.52)	0.55 (0.54 - 0.55)	0.54 (0.53 - 0.55)	0.58 (0.58 - 0.59)	0.57 (0.56 - 0.59)	0.45 (0.44 - 0.46)	0.44 (0.42 - 0.46)

sensitivity	0.95 (0.95 - 0.96)	0.95 (0.94 - 0.95)	0.90 (0.89 - 0.90)	0.89 (0.88 - 0.90)	0.78 (0.77 - 0.79)	0.77 (0.75 - 0.78)	0.35 (0.34 - 0.36)	0.34 (0.32 - 0.36)
specificity	0.39 (0.38 - 0.39)	0.38 (0.37 - 0.39)	0.52 (0.52 - 0.53)	0.52 (0.51 - 0.53)	0.69 (0.68 - 0.69)	0.69 (0.68 - 0.70)	0.93 (0.93 - 0.94)	0.93 (0.93 - 0.94)
precision	0.35 (0.35 - 0.36)	0.34 (0.33 - 0.35)	0.40 (0.39 - 0.40)	0.39 (0.38 - 0.40)	0.46 (0.46 - 0.47)	0.46 (0.45 - 0.47)	0.64 (0.63 - 0.65)	0.63 (0.61 - 0.65)
c-statistic	0.81 (0.80 - 0.81)	0.80 (0.79 - 0.81)	0.81 (0.80 - 0.81)	0.80 (0.79 - 0.81)	0.81 (0.80 - 0.81)	0.80 (0.79 - 0.81)	0.81 (0.80 - 0.81)	0.80 (0.79 - 0.81)
pr_auc	0.58 (0.57 - 0.59)	0.56 (0.54 - 0.58)	0.58 (0.57 - 0.59)	0.56 (0.54 - 0.58)	0.58 (0.57 - 0.59)	0.56 (0.54 - 0.58)	0.58 (0.57 - 0.59)	0.56 (0.54 - 0.58)
brier	0.15 (0.15 - 0.15)	0.15 (0.15 - 0.15)	0.15 (0.15 - 0.15)	0.15 (0.15 - 0.15)	0.15 (0.15 - 0.15)	0.15 (0.15 - 0.15)	0.15 (0.15 - 0.15)	0.15 (0.15 - 0.15)
calibration_slope	1.07 (1.05 - 1.09)	1.02 (0.99 - 1.06)	1.07 (1.05 - 1.09)	1.02 (0.99 - 1.06)	1.07 (1.05 - 1.09)	1.02 (0.99 - 1.06)	1.07 (1.05 - 1.09)	1.02 (0.99 - 1.06)
calibration_intercept	-0.01 (-0.01 - -0.00)	0.00 (-0.00 - 0.01)	-0.01 (-0.01 - -0.00)	0.00 (-0.00 - 0.01)	-0.01 (-0.01 - -0.00)	0.00 (-0.00 - 0.01)	-0.01 (-0.01 - -0.00)	0.00 (-0.00 - 0.01)

Supplementary Table XXI. An overview of all performance metrics for 30-day mortality with different thresholds (Logistic regression)

Logistic regression 30-day mortality								
Metric	Threshold 0.1 Train Data	Threshold 0.1 Test Data	Threshold 0.15 Train Data	Threshold 0.15 Test Data	Threshold 0.25 Train Data	Threshold 0.25 Test Data	Threshold 0.5 Train Data	Threshold 0.5 Test Data
f1	0.33 (0.33 - 0.34)	0.33 (0.31 - 0.34)	0.37 (0.36 - 0.38)	0.36 (0.34 - 0.38)	0.34 (0.33 - 0.35)	0.35 (0.33 - 0.38)	0.08 (0.07 - 0.09)	0.09 (0.07 - 0.11)
sensitivity	0.76 (0.75 - 0.78)	0.77 (0.74 - 0.79)	0.61 (0.60 - 0.62)	0.62 (0.59 - 0.64)	0.34 (0.33 - 0.35)	0.36 (0.34 - 0.39)	0.05 (0.04 - 0.05)	0.05 (0.04 - 0.06)
specificity	0.72 (0.71 - 0.72)	0.72 (0.71 - 0.72)	0.83 (0.83 - 0.83)	0.83 (0.82 - 0.83)	0.93 (0.93 - 0.94)	0.93 (0.93 - 0.94)	0.99 (0.99 - 1.00)	0.99 (0.99 - 1.00)
precision	0.21 (0.21 - 0.22)	0.21 (0.20 - 0.22)	0.26 (0.26 - 0.27)	0.26 (0.24 - 0.27)	0.34 (0.33 - 0.35)	0.34 (0.32 - 0.37)	0.47 (0.43 - 0.51)	0.48 (0.40 - 0.56)
c-statistic	0.82 (0.81 - 0.82)	0.82 (0.81 - 0.83)	0.82 (0.81 - 0.82)	0.82 (0.81 - 0.83)	0.82 (0.81 - 0.82)	0.82 (0.81 - 0.83)	0.82 (0.81 - 0.82)	0.82 (0.81 - 0.83)
pr_auc	0.30 (0.29 - 0.32)	0.30 (0.28 - 0.33)	0.30 (0.29 - 0.32)	0.30 (0.28 - 0.33)	0.30 (0.29 - 0.32)	0.30 (0.28 - 0.33)	0.30 (0.29 - 0.32)	0.30 (0.28 - 0.33)
brier	0.07 (0.07 - 0.07)	0.07 (0.07 - 0.07)	0.07 (0.07 - 0.07)	0.07 (0.07 - 0.07)	0.07 (0.07 - 0.07)	0.07 (0.07 - 0.07)	0.07 (0.07 - 0.07)	0.07 (0.07 - 0.07)

calibration_slope	0.99 (0.95 - 1.02)	0.97 (0.90 - 1.03)	0.99 (0.95 - 1.02)	0.97 (0.90 - 1.03)	0.99 (0.95 - 1.02)	0.97 (0.90 - 1.03)	0.99 (0.95 - 1.02)	0.97 (0.90 - 1.03)
calibration_intercept	-0.00 (-0.01 - - 0.00)	-0.01 (-0.01 - - 0.00)	-0.00 (-0.01 - - 0.00)	-0.01 (-0.01 - - 0.00)	-0.00 (-0.01 - - 0.00)	-0.01 (-0.01 - - 0.00)	-0.00 (-0.01 - - 0.00)	-0.01 (-0.01 - -0.00)

Supplementary Table XXII. An overview of all performance metrics for 90-day mortality with different thresholds (Logistic regression)

Logistic regression 90-day mortality								
Metric	Threshold 0.1 Train Data	Threshold 0.1 Test Data	Threshold 0.15 Train Data	Threshold 0.15 Test Data	Threshold 0.25 Train Data	Threshold 0.25 Test Data	Threshold 0.5 Train Data	Threshold 0.5 Test Data
f1	0.40 (0.39 - 0.40)	0.40 (0.39 - 0.41)	0.44 (0.43 - 0.44)	0.44 (0.43 - 0.45)	0.45 (0.44 - 0.46)	0.46 (0.44 - 0.48)	0.23 (0.22 - 0.24)	0.24 (0.22 - 0.26)
sensitivity	0.87 (0.86 - 0.88)	0.88 (0.87 - 0.90)	0.76 (0.75 - 0.77)	0.77 (0.76 - 0.79)	0.53 (0.52 - 0.54)	0.56 (0.54 - 0.58)	0.15 (0.14 - 0.16)	0.15 (0.14 - 0.17)
specificity	0.56 (0.56 - 0.57)	0.55 (0.55 - 0.56)	0.70 (0.70 - 0.71)	0.69 (0.68 - 0.70)	0.85 (0.85 - 0.86)	0.85 (0.84 - 0.85)	0.98 (0.98 - 0.98)	0.98 (0.98 - 0.98)
precision	0.26 (0.25 - 0.26)	0.26 (0.25 - 0.27)	0.31 (0.30 - 0.31)	0.31 (0.30 - 0.32)	0.39 (0.38 - 0.40)	0.39 (0.37 - 0.41)	0.56 (0.54 - 0.58)	0.56 (0.52 - 0.60)
c-statistic	0.81 (0.80 - 0.81)	0.81 (0.80 - 0.82)	0.81 (0.80 - 0.81)	0.81 (0.80 - 0.82)	0.81 (0.80 - 0.81)	0.81 (0.80 - 0.82)	0.81 (0.80 - 0.81)	0.81 (0.80 - 0.82)
pr_auc	0.41 (0.40 - 0.42)	0.42 (0.39 - 0.44)	0.41 (0.40 - 0.42)	0.42 (0.39 - 0.44)	0.41 (0.40 - 0.42)	0.42 (0.39 - 0.44)	0.41 (0.40 - 0.42)	0.42 (0.39 - 0.44)
brier	0.10 (0.10 - 0.11)	0.11 (0.10 - 0.11)	0.10 (0.10 - 0.11)	0.11 (0.10 - 0.11)	0.10 (0.10 - 0.11)	0.11 (0.10 - 0.11)	0.10 (0.10 - 0.11)	0.11 (0.10 - 0.11)
calibration_slope	0.99 (0.96 - 1.01)	0.98 (0.94 - 1.03)	0.99 (0.96 - 1.01)	0.98 (0.94 - 1.03)	0.99 (0.96 - 1.01)	0.98 (0.94 - 1.03)	0.99 (0.96 - 1.01)	0.98 (0.94 - 1.03)
calibration_intercept	-0.00 (-0.00 - 0.00)	-0.00 (-0.01 - 0.00)	-0.00 (-0.00 - 0.00)	-0.00 (-0.01 - 0.00)	-0.00 (-0.00 - 0.00)	-0.00 (-0.01 - 0.00)	-0.00 (-0.00 - 0.00)	-0.00 (-0.01 - 0.00)

Supplementary Table XXIII. An overview of all performance metrics for 1-year mortality with different thresholds (Logistic regression)

Logistic regression 1-year mortality								
Metric	Threshold 0.1 Train Data	Threshold 0.1 Test Data	Threshold 0.15 Train Data	Threshold 0.15 Test Data	Threshold 0.25 Train Data	Threshold 0.25 Test Data	Threshold 0.5 Train Data	Threshold 0.5 Test Data
f1	0.50 (0.50 - 0.51)	0.50 (0.49 - 0.51)	0.54 (0.53 - 0.54)	0.54 (0.52 - 0.55)	0.57 (0.56 - 0.58)	0.57 (0.56 - 0.58)	0.46 (0.45 - 0.47)	0.45 (0.44 - 0.47)

sensitivity	0.95 (0.95 - 0.96)	0.95 (0.94 - 0.96)	0.90 (0.89 - 0.90)	0.89 (0.88 - 0.90)	0.75 (0.75 - 0.76)	0.75 (0.74 - 0.77)	0.37 (0.36 - 0.38)	0.36 (0.34 - 0.38)
specificity	0.36 (0.35 - 0.36)	0.35 (0.34 - 0.36)	0.50 (0.49 - 0.50)	0.50 (0.49 - 0.51)	0.69 (0.69 - 0.69)	0.70 (0.69 - 0.71)	0.92 (0.92 - 0.92)	0.92 (0.92 - 0.93)
precision	0.34 (0.34 - 0.35)	0.34 (0.33 - 0.35)	0.38 (0.38 - 0.39)	0.38 (0.37 - 0.39)	0.46 (0.45 - 0.46)	0.46 (0.45 - 0.47)	0.62 (0.61 - 0.63)	0.61 (0.59 - 0.64)
c-statistic	0.80 (0.79 - 0.80)	0.80 (0.79 - 0.81)	0.80 (0.79 - 0.80)	0.80 (0.79 - 0.81)	0.80 (0.79 - 0.80)	0.80 (0.79 - 0.81)	0.80 (0.79 - 0.80)	0.80 (0.79 - 0.81)
pr_auc	0.57 (0.56 - 0.58)	0.56 (0.55 - 0.58)	0.57 (0.56 - 0.58)	0.56 (0.55 - 0.58)	0.57 (0.56 - 0.58)	0.56 (0.55 - 0.58)	0.57 (0.56 - 0.58)	0.56 (0.55 - 0.58)
brier	0.15 (0.15 - 0.15)	0.15 (0.15 - 0.15)	0.15 (0.15 - 0.15)	0.15 (0.15 - 0.15)	0.15 (0.15 - 0.15)	0.15 (0.15 - 0.15)	0.15 (0.15 - 0.15)	0.15 (0.15 - 0.15)
calibration_slope	1.00 (0.98 - 1.01)	0.99 (0.95 - 1.02)	1.00 (0.98 - 1.01)	0.99 (0.95 - 1.02)	1.00 (0.98 - 1.01)	0.99 (0.95 - 1.02)	1.00 (0.98 - 1.01)	0.99 (0.95 - 1.02)
calibration_intercept	-0.00 (-0.01 - 0.00)	-0.00 (-0.01 - 0.01)	-0.00 (-0.01 - 0.00)	-0.00 (-0.01 - 0.01)	-0.00 (-0.01 - 0.00)	-0.00 (-0.01 - 0.01)	-0.00 (-0.01 - 0.00)	-0.00 (-0.01 - 0.01)

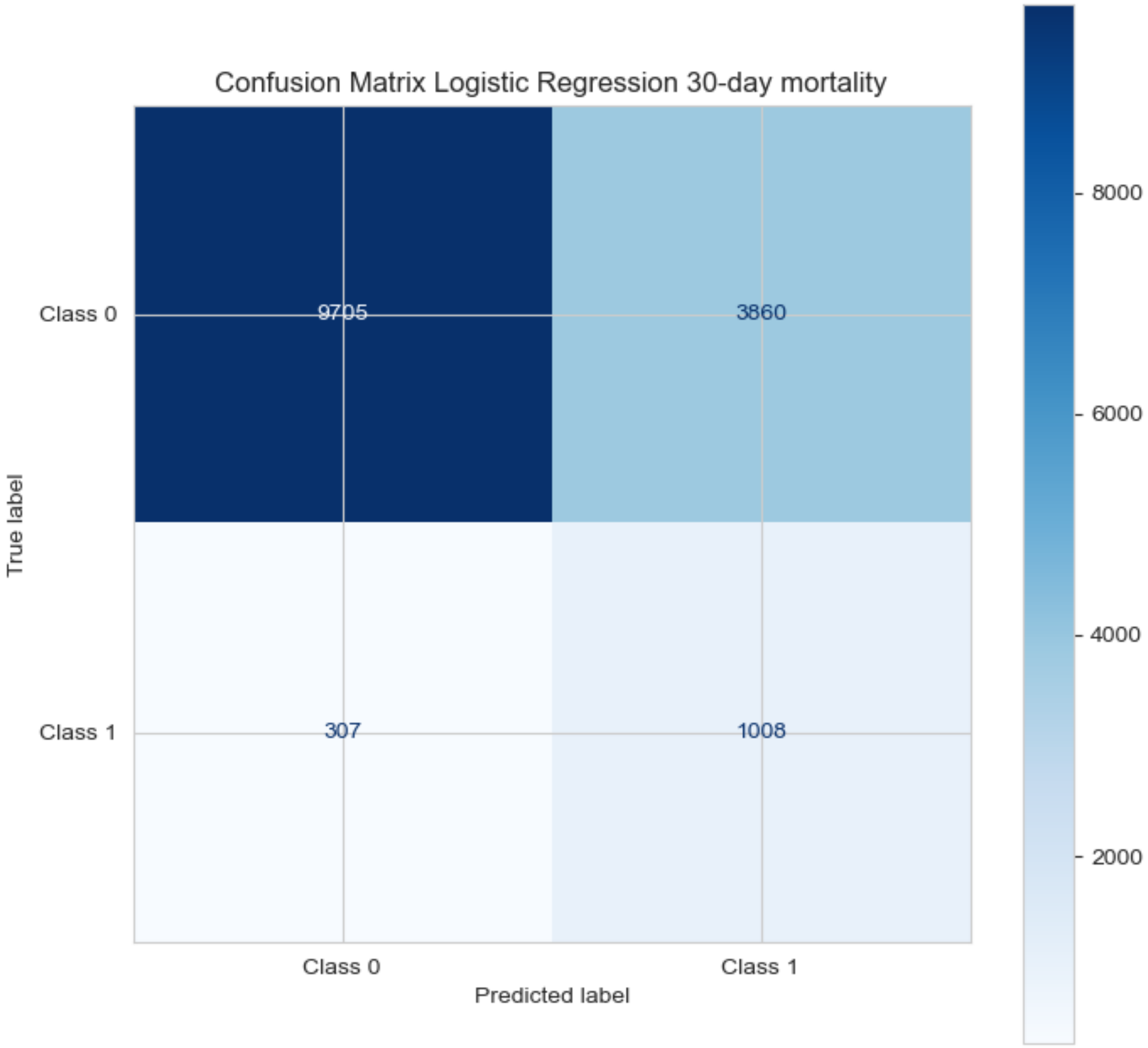

Supplementary Figure I. Confusion matrix for 30-day mortality prediction using the final best performing algorithm (Logistic regression) using the threshold 0.10.

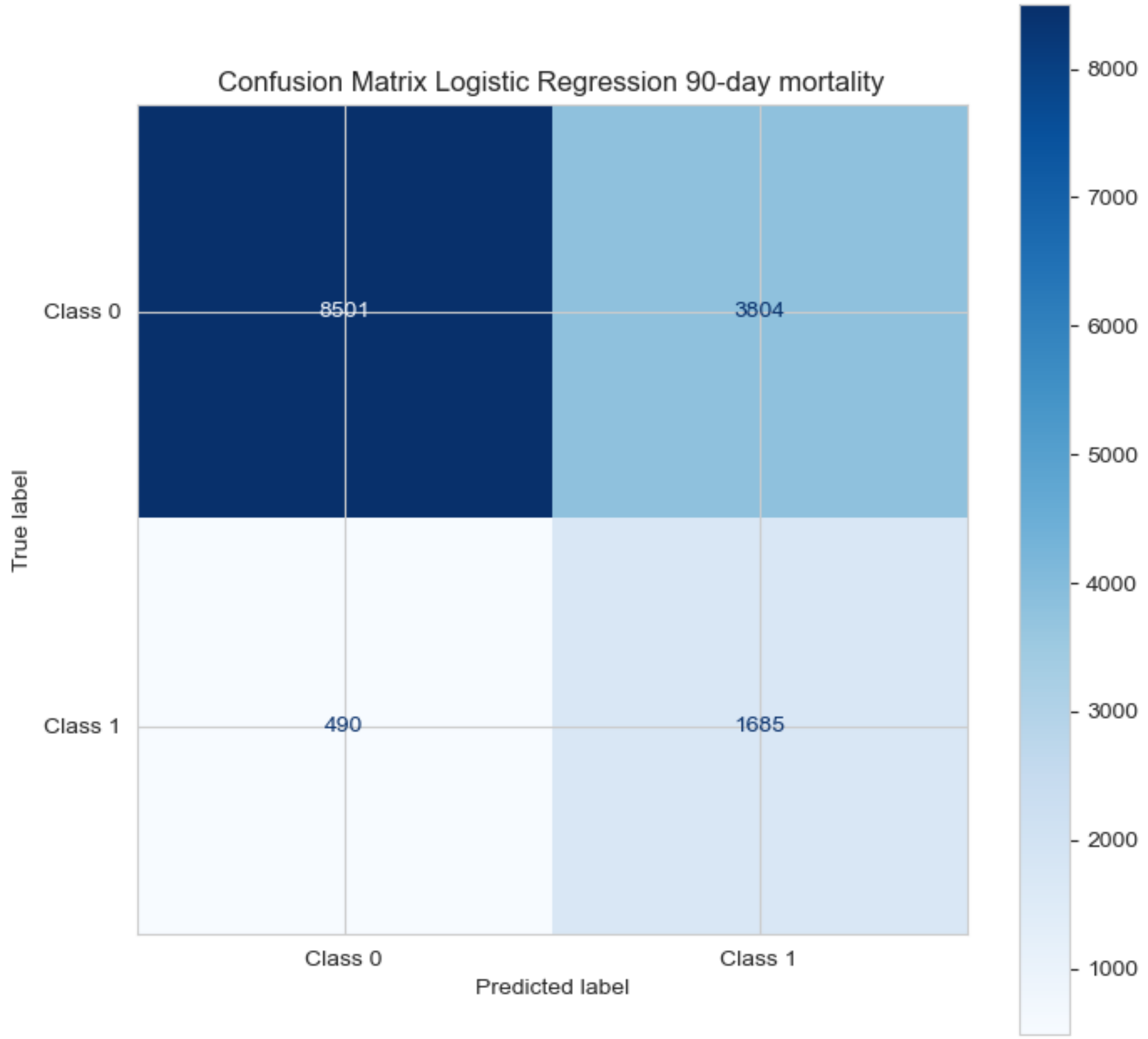

Supplementary Figure II. Confusion matrix for 90-day mortality prediction using the final best performing algorithm (Logistic regression) using the threshold 0.15.

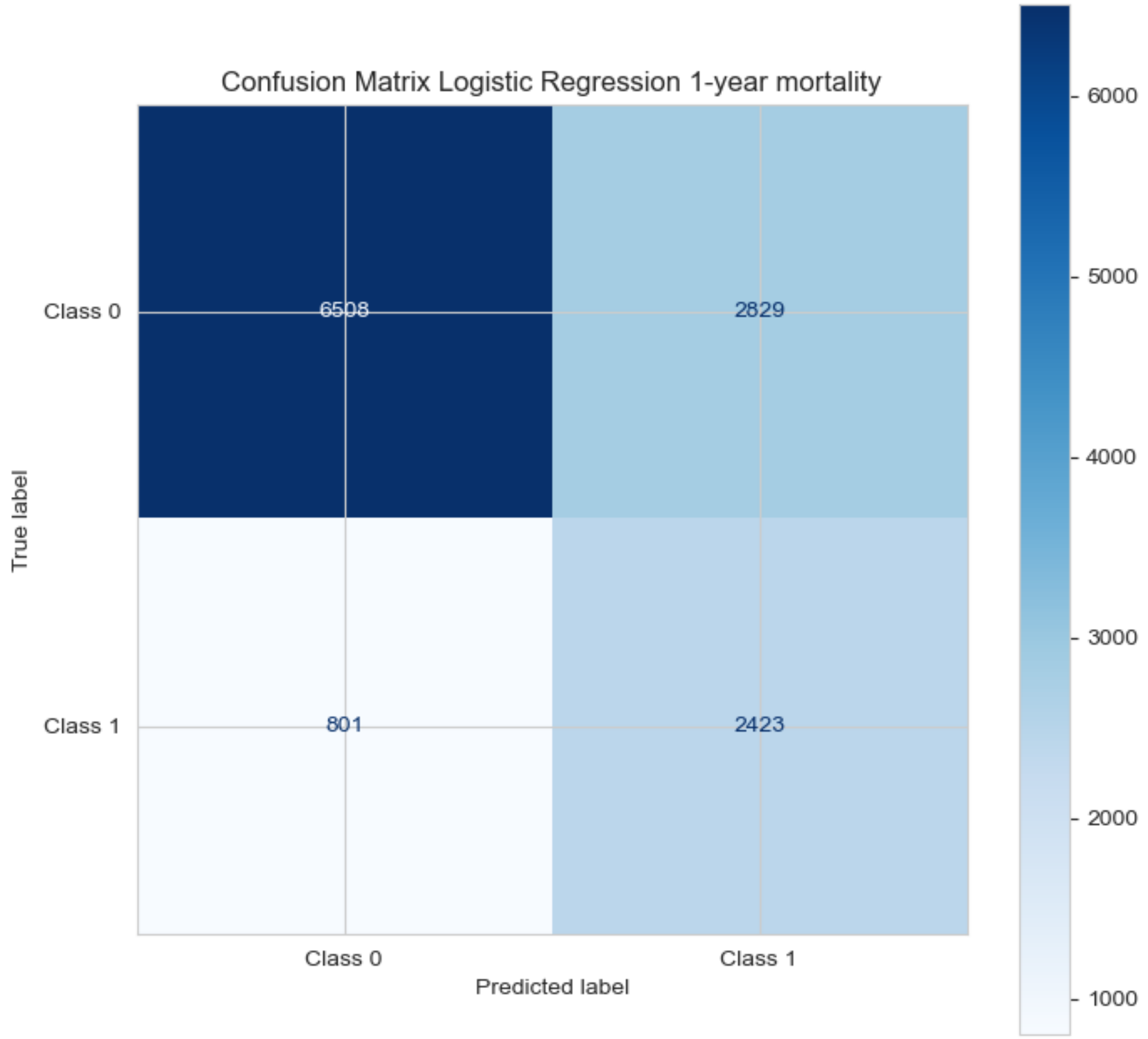

Supplementary Figure III. Confusion matrix for 1-year mortality prediction using the final best performing algorithm (Logistic regression) using the threshold 0.25.

References

1. Gravesteijn BY, Sewalt CA, Venema E, Nieboer D, Steyerberg EW. Missing Data in Prediction Research: A Five-Step Approach for Multiple Imputation, Illustrated in the CENTER-TBI Study. J Neurotrauma 2021; 38: 1842–1857.

2. Stekhoven DJ, Bühlmann P. Missforest-Non-parametric missing value imputation for mixed-type data. Bioinformatics 2012; 28: 112–118.

3. Wainer J. Comparison of 14 different families of classification algorithms on 115 binary datasets. 2016;

4. Maroco J, Silva D, Rodrigues A, Guerreiro M, Santana I, De Mendonça A. Data mining methods in the prediction of Dementia: A real-data comparison of the accuracy, sensitivity and specificity of linear discriminant analysis, logistic regression, neural networks, support vector machines, classification trees and random forests. BMC Res Notes 2011; 4: 299.

5. Fernández-Delgado M, Cernadas E, Barro S, Amorim D. Do we need hundreds of classifiers to solve real world classification problems? J Mach Learn Res 2014; 15: 3133–3181.

6. Luo W, Phung D, Tran T *et al.* Guidelines for developing and reporting machine learning predictive models in biomedical research: A multidisciplinary view. J Med Internet Res 2016; 18: 1–10.

7. Collins GS, Reitsma JB, Altman DG, Moons KGM. Transparent reporting of a multivariable prediction model for individual prognosis or diagnosis (TRIPOD): the TRIPOD statement. BMJ 2015; 350: 7594.

8. Steyerberg EW, Vergouwe Y. Towards better clinical prediction models: Seven steps for development and an ABCD for validation. Eur Heart J 2014; 35: 1925–1931.

9. Van Calster B, Vickers AJ. Calibration of risk prediction models: Impact on decision-analytic performance. Med Decis Mak 2015; 35: 162–169.

10. Steyerberg EW, Vickers AJ, Cook NR *et al.* Assessing the performance of prediction models: A framework for traditional and novel measures. Epidemiology 2010; 21: 128–138.
